# Behavioral and Neural Indices of Metacognitive Sensitivity in Preverbal Infants

**DOI:** 10.1016/j.cub.2016.09.004

**Published:** 2016-11-21

**Authors:** Louise Goupil, Sid Kouider

**Affiliations:** 1 Brain and Consciousness Group, CNRS, École Normale Supérieure, PSL Research University, 75005 Paris, France; 2 Ecole Doctorale Cerveau Cognition Comportement, Université Pierre et Marie Curie, 75005 Paris, France; 3 Science Division, Department of Psychology, New York University Abu Dhabi, Saadiyat Island, PO Box 129188, Abu Dhabi, UAE

**Keywords:** EEG, cognitive neuroscience, infants, metacognition, confidence, error monitoring

## Abstract

Humans adapt their behavior not only by observing the consequences of their actions but also by internally monitoring their performance. This capacity, termed metacognitive sensitivity [1, 2], has traditionally been denied to young children because they have poor capacities in verbally reporting their own mental states [3, 4, 5]. Yet, these observations might reflect children’s limited capacities for explicit self-reports, rather than limitations in metacognition per se. Indeed, metacognitive sensitivity has been shown to reflect simple computational mechanisms [1, 6, 7, 8], and can be found in various non-verbal species [7, 8, 9, 10]. Thus, it might be that this faculty is present early in development, although it would be discernible through implicit behaviors and neural indices rather than explicit self-reports. Here, by relying on such non-verbal indices, we show that 12- and 18-month-old infants internally monitor the accuracy of their own decisions. At the behavioral level, infants showed increased persistence in their initial choice after making a correct as compared to an incorrect response, evidencing an appropriate evaluation of decision confidence. Moreover, infants were able to use decision confidence adaptively to either confirm their initial choice or change their mind. At the neural level, we found that a well-established electrophysiological signature of error monitoring in adults, the error-related negativity, is similarly elicited when infants make an incorrect choice. Hence, although explicit forms of metacognition mature later during childhood, infants already estimate decision confidence, monitor their errors, and use these metacognitive evaluations to regulate subsequent behavior.

## Highlights

• Preverbal infants internally monitor the accuracy of their own decisions
• Infants’ post-decision persistence reflects decision confidence
• Infants can rely on decision confidence to regulate subsequent behavior
• After errors, a typical EEG marker of performance monitoring is elicited

## Teaser

Goupil and Kouider studied implicit behaviors and neural indices in infants and show that they have metacognition. Infants showed more post-decision persistence for correct compared to incorrect choices, evidencing an evaluation of decision confidence. After a mistake, infants elicited the same neuronal signature of error monitoring as in adults.

## Results

Here we tested whether two core metacognitive processes, decision confidence and error monitoring, are already present in infancy. We developed a non-verbal procedure in which 18- and 12-month-old infants made a binary decision that allowed measuring performance at the cognitive (i.e., first-order) level. Post-decision measurements then allowed assessing infants’ metacognitive (i.e., second-order) abilities. For confidence (experiments 1–3), we relied on a behavioral measure reflecting infants’ willingness to search for a reward depending on the accuracy of their decision. Studies in rats suggest that similar measures of post-decision persistence reflect a metacognitive computation of confidence [7, 11]. More precisely, rats’ willingness to wait for a reward can be accounted for by a simple model in which confidence is estimated by comparing the accumulated evidence against the decision bound [7]. Importantly, this process involves the orbito-frontal cortex, a region distinct from areas involved in first-order decisions [12] but analogous to areas involved in metacognition in humans [13]. For error detection (experiment 3), we relied on a neural marker well documented in adults. Specifically, by probing for the presence of the error-related negativity (ERN), an electroencephalography (EEG) component observed whenever subjects make an error [1, 14, 15], we tested whether the mechanisms responsible for error monitoring in adults are already functional in 12-month-old infants.

### 18-Month-Old Infants Monitor Decision Confidence

**Figure 1:**
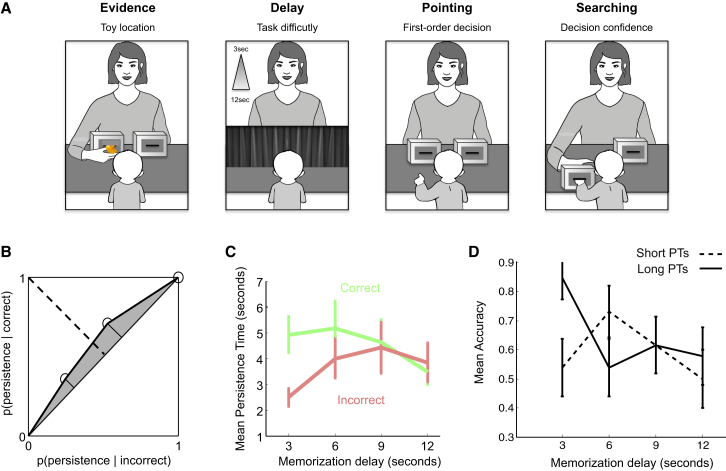
Experiment 1 (A) Experimental design. During the familiarization phase (four trials), the infant saw the experimenter hide a toy in one of two opaque boxes. The experimenter then asked her to point to indicate where she remembered the toy to be. As soon as the infant produced a pointing response, the selected box was pushed forward to allow retrieving the toy. The test phase (eight trials) was similar except for two aspects. First, a variable memorization delay (3, 6, 9, or 12 s) was introduced, during which a curtain was closed to occlude the boxes from the infant’s view. Second, unbeknownst to the child, the toys were now out of reach during the searching period (i.e., hidden within an unreachable pocket inside the box), so as to measure infants’ persistence. As soon as infants gave up searching, the experimenter recovered the toy from the box and showed it to the infant before either (1) replacing it in the box and letting the infant recover it after a correct response, or (2) starting a new trial when no response or an incorrect response was given by the infant. (B) Mean type 2 ROC curve. Individual type 2 ROC curves were constructed by plotting the probability of searching for a certain amount of time for correct trials against the probability of searching for an equivalent amount of time for incorrect trials, cumulated across persistence time (PT) terciles. The gray area between the ROC curve and the diagonal is an estimate of the extent to which infants searched longer for correct versus incorrect trials: a type 2 ROC curve departing upward from the diagonal indicates that participants were more likely to search longer for correct over incorrect trials. The dashed line represents the minor diagonal; the solid thick line corresponds to the ROC curve; and the thin solid line corresponds to the major diagonal. Area under the ROC curve is shown in gray. (C) Relationship between PTs and accuracy depending on task difficulty. PTs were averaged separately for correct and incorrect trials for each level of difficulty. (D) Mean accuracy as a function of task difficulty, computed separately for short (<median) and long (>median) PTs. Although PTs did not significantly predict accuracy when all conditions were collapsed (χ^2^ = 2.54; p = 0.1), there was a significant interaction between PTs and delay (χ^2^ = 6.34; p < 0.02). In the 3-s condition, accuracy was lower for short PTs as compared to long PTs (t(25) = 2.85; p < 0.01). Importantly, the accuracy for short PTs did not differ from chance level (t(25) = 0.38; p > 0.7), whereas the accuracy for long PTs was greatly above chance (t(25) = 4.79; p < 0.001). Mean accuracy did not differ from chance level even when dividing PTs in deciles and considering only the tenth-shortest PTs (t(25) = 0.4; p > 0.7). Error bars show SEMs. See also Figure S1.

In experiment 1, 18-month-old infants (n = 29) saw an object being hidden in one of two opaque boxes and, after a delay, were asked to point to indicate where the object was concealed (see Figure 1A and the Supplemental Experimental Procedures). First-order performance on this task was assessed along a parametric variation of difficulty (i.e., memorizing the location of the toy for a brief or longer delay). Immediately following this choice, infants were provided with the selected box. The amount of time they were willing to search within this box before giving up was used as a measure of post-decision persistence. Importantly, persistence times (PTs) were measured in the absence of any external feedback on performance, allowing us to use this measure as a proxy for confidence [7].

Infants pointed toward the correct box with above chance-level accuracy (t(28) = 4.9; p < 0.001) but experienced more difficulty as memorization delay increased: accuracy was significantly above chance level for the shortest delay of 3 s (t(25) = 2.81; p < 0.01) but not for any of the longer delays (all p values > 0.06; Figure S1). We then examined whether infants’ persistence in their initial choice correlated with accuracy. Consistent with our hypothesis, we observed that infants searched longer in the box following correct as compared to incorrect decisions (t(28) = 2.1; p < 0.05). To ensure that our results were not contaminated by individual biases in searching more or less in the box (e.g., liberal versus conservative profiles), we performed a complementary non-parametric and bias-free analysis [16, 17]. PT terciles were computed to approximate a three-point confidence scale and construct individual type 2 receiver operating characteristic (ROC) curves [16, 17] (Figure 1B and the Supplemental Experimental Procedures). Mean area under the type 2 ROC curves was significantly greater than chance level (t(28) = 2.09; p < 0.03), suggesting that, independent of individual searching biases, infants persisted more in their choices after a correct as compared to an incorrect choice.

Although this result suggests that infants displayed metacognitive sensitivity, a potential concern might be that infants’ persistence reflected the strength of the memory trace used to perform the initial choice (i.e., a first-order decision variable), rather than a metacognitive evaluation (i.e., a second-order monitoring of the decision variable). More precisely, it might be that infants simply search longer when their memory about the toy location is strong. Given that accuracy also depends on this memory trace, a spurious correlation between accuracy and persistence might also be expected in this case. Our manipulation of the memorization delay was specifically introduced to disentangle these two interpretations. Indeed, a first-order interpretation predicts a direct correlation between PTs and delay, because the memory trace allegedly fades out with time. By contrast, a metacognitive interpretation predicts that (1) persistence should depend on accuracy, as long as a reliable memory trace is available (i.e., for above chance-level performance), and (2) that the correlation between persistence and accuracy should vanish when no reliable memory trace is available (i.e., for chance-level performance) (see [7] for a computational account). Consistent with this last interpretation, we found an interaction between delay and accuracy (χ^2^ = 8.97; p *<* 0.03; likelihood ratio test; mixed models were used because of missing cells; see the Supplemental Experimental Procedures) but no main effect of memorization delay (χ^2^ = 3.16; p > 0.3). This interaction was due to infants searching less following incorrect as compared to correct trials only when accuracy was above chance (i.e., for the shortest delay of 3 s; χ^2^ = 10; p *<* 0.01; see Figure 1C).

Another signature of confidence is that performance should fall to chance level for the lowest levels of confidence (i.e., here for the shortest PTs). Thus, we also examined accuracy as a function of PTs and memorization delay (see Figure 1D). A mixed logistic regression revealed a significant interaction (χ^2^ = 6.34; p < 0.02), reflecting the fact that PTs significantly predicted accuracy in the easy condition (i.e., at 3 s: χ^2^ = 7.8; p < 0.01), but not at any of the longer delays (all p values > 0.3). Interestingly, the intercept of the regression did not differ from chance level (estimate: −0.14 ± 0.13; p > 0.2). Thus, short PTs do not predict accuracy below 50% but rather correspond to chance-level performance, as would be expected for a true marker of confidence. This result also suggests that PTs reflect confidence rather than error monitoring. Indeed, for error monitoring, below chance-level accuracy would be expected for the lowest level of persistence.

**Figure 2:**
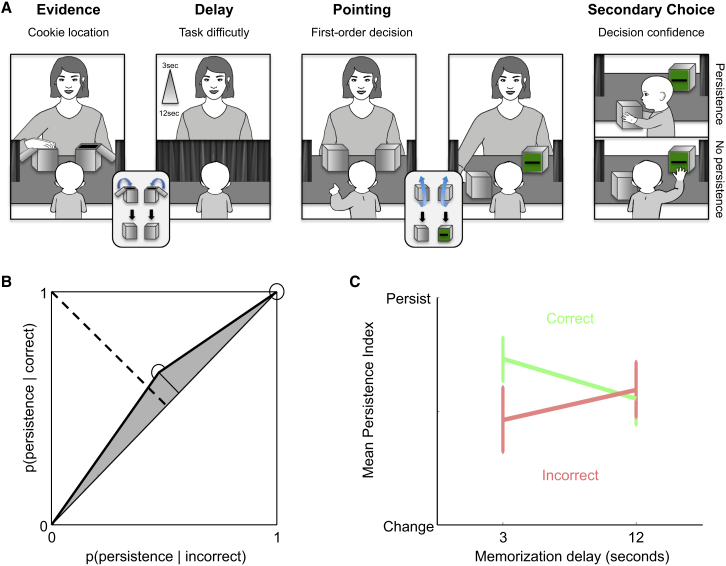
Experiment 2 (A) Experimental design. The two boxes contained a lid, which could only be opened by an adult. Although one of them had a slit such that infants were able to directly reach for its content (unsealed box), there was no slit in the other box, rendering its content unreachable without the help of an adult (sealed box). Importantly, both boxes looked identical from the infants’ point of view, such that they could not know which box they were selecting (sealed versus unsealed box). During the familiarization phase (four trials), infants saw the experimenter hide a biscuit in one of two boxes. The experimenter then asked them to point to indicate where they remembered the biscuit to be. As soon as infants produced a pointing response, the selected box was pushed forward. During the first two trials, the biscuit was hidden in the unsealed box, so infants could directly recover it. During the last two trials, the biscuit was hidden in the sealed box, so as to teach infants to ask their caregiver to open it for them. The test phase (eight trials) was similar to the familiarization phase except for two elements. First, a variable memorization delay (3 or 12 s) was introduced. Second, the biscuits were now hidden half of the time in the unsealed box and the other half in the sealed box. Importantly, infants selected the sealed and unsealed boxes equally often (t(21) = 0.3; p > 0.7), showing that they could not discriminate the two boxes before pointing. Selection of the sealed box forced infants to either confirm their initial choice by asking their caregiver to open it or invalidate their initial choice by turning to the alternative box, whose content was directly reachable. (B) Mean type 2 ROC curve. Individual type 2 ROC curves were constructed by plotting the probability of producing one type of secondary response for correct trials against the probability of producing the same type of secondary response for incorrect trials. (C) Relationship between persistence and accuracy depending on task difficulty. A persistence index was obtained for each subject by coding secondary actions in a binary fashion, with zeros corresponding to changes of mind (i.e., no persistence) and ones corresponding to asking for help (i.e., persistence). Persistence indices were then averaged separately for correct and incorrect trials, per levels of difficulty, for each participant. Error bars show SEMs. See also Figure S2.

Our results are thus consistent with a metacognitive interpretation according to which infants’ post-decision persistence reflects an internal—second-order—evaluation of their decision (see the Supplemental Experimental Procedures for additional discussion). Another test would be to show that infants not only persist differentially in their choices depending on accuracy but also use the output of this monitoring process to adjust behavior. In other words, if we observe that infants are able to regulate subsequent choices depending on an evaluation of a first—distinct—decision, we can infer that some form of metacognitive evaluation has taken place. In experiment 2, we thus tested whether infants can rely on confidence to appropriately select a secondary action that is totally distinct from the pointing response. Whereas the first-order task was identical to experiment 1, the metacognitive task consisted of deciding whether to confirm or invalidate the initial choice (see Figure 2A). After selecting one of the two boxes by pointing, infants received, in half of the trials, a box that they could not open by themselves. In order to recover its content, infants were consequently forced to either persist in their choice by asking their caregiver to open the selected box or revise their choice by reaching toward the alternative box. We predicted that infants’ choice to persist or change their mind should depend on the accuracy of their initial decision. In addition, the relationship between first-order (pointing choice accuracy) and second-order (confirming or revising their initial choice) variables should vary with the difficulty of the task (memorization delay).

### 18-Month-Old Infants Use Decision Confidence to Regulate Subsequent Behavior

Infants’ (n = 22) pointing accuracy was significantly above chance level for the delay of 3 s (t(21) = 2.9; p < 0.01), but not for the delay of 12 s (t(21) = 1.4; p > 0.17) (see Figure S2). Infants always selected one of the two possible second-order actions after making a pointing response. Thus, a two-point confidence scale was obtained by dummy coding “ask for help” responses as ones (i.e., persistence) and “changes of mind” as zeros (i.e., no persistence). This persistence index was then used to construct individual type 2 ROC curves to test whether infants’ choice of a second action depended on accuracy (Figure 2B). Mean area under the type 2 ROC curves was significantly greater than chance level (t(21) = 2.26; p < 0.04), showing that infants persisted more in their initial choice after correct compared to incorrect responses. Examining the impact of task difficulty on this effect revealed an interaction between delay and accuracy (χ^2^ = 11.25; p *<* 0.001; see Figure 2C) but no significant effect of delay (χ^2^ = 0.48; p *>* 0.4). This interaction reflected the fact that the impact of accuracy on the second-order measure was significant only for the shortest delay of 3 s (χ^2^ = 13.61; p < 0.001). Thus, infants’ probability of asking for help versus changing their mind was determined by the accuracy of their initial choice, but only when the memorization delay allowed for above chance-level performance. These results demonstrate that infants can rely on an internal evaluation of confidence in order to regulate subsequent behavior.

Our experiments reveal behavioral indices of decision confidence in infants. Yet it remains unclear whether such metacognitive evaluations rely on the same mechanisms as those used by adults when reporting confidence. To examine further the mechanisms underlying metacognitive sensitivity in infancy, we turned to error monitoring. Indeed, the neural computations underlying this second metacognitive process are well documented in adults [1, 18]. By probing for comparable neural signatures in infants, one might thus demonstrate shared mechanisms across the two populations. A particularly interesting signature is the ERN, an event-related potential observed over fronto-central electrodes whenever subjects make an error [1, 14, 15]. Studies in human adults show that the ERN relates to post-error adjustments and error-correcting activity [12, 19] and originates in the anterior cingulate cortex [1, 20]. They also suggest that this component reflects mechanisms of performance monitoring that function by comparing the response that should have been made with the response that was actually performed [1, 14, 21, 22].

An equivalent of the ERN has been documented when 6- and 9-month-old infants perceive an unsuspected event in their environment (i.e., a conflict between expected and actual events) [23]. More precisely, this component was evoked by simple numerical violations, and thus was driven by sensory information in the external environment. An important question is whether an ERN can be generated in infants in response to their own error (i.e., following a conflict between required and actual responses) [22]. This finding would demonstrate that infants monitor their own errors in the absence of any external feedback, by relying on internal, metacognitive computations.

### Behavioral Indices of Decision Confidence and Neural Signatures of Error Monitoring in 12-Month-Old Infants

**Figure 3:**
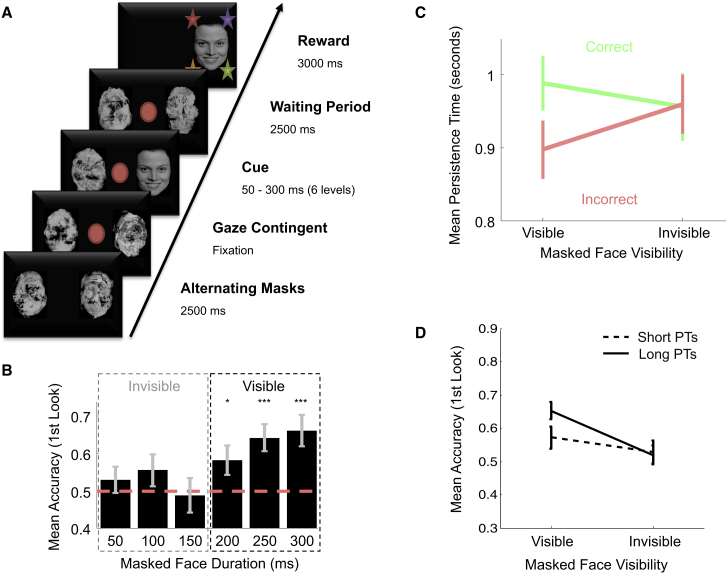
Experiment 3 (A) Experimental design. EEG and eye movements were recorded while infants were presented with a masked-face detection paradigm. As soon as the infant looked at the central fixation (red circle), a face was presented for variable durations (50–300 ms in steps of 50 ms) to the left or right side of the screen. A waiting period then followed, in which nothing but backward masks and the fixation remained on the screen. During this period, the first look toward one or the other side of the screen was taken as the first-order response, and the consecutive time the infant was willing to wait in the selected frame after the initial saccade was taken as post-decision persistence. Following the waiting period, the face reappeared on the same side for 3 s, now as a reward (i.e., along with music and blinking, multi-colored stars). (B) Mean accuracy of first looks depending on the duration of the masked face. The red dashed line represents chance level. ^∗^p < 0.05; ^∗∗∗^p < 0.001. (C) Relationship between PTs and first-order accuracy depending on visibility. PTs were averaged separately for correct and incorrect trials for each level of visibility. (D) Mean accuracy as a function of task difficulty, computed separately for short (<median) and long (>median) PTs. There was a marginal interaction between PTs and task difficulty (F(1,54) = 3.7; p = 0.06), reflecting the fact that PTs significantly predicted accuracy in the visible (F(1,54) = 6; p < 0.02) but not in the invisible condition (p > 0.7). Post hoc tests on visible trials further revealed that accuracy was marginally lower for short PTs as compared to long PTs (t(54) = 1.97; p = 0.06), and that the accuracy for short PTs remained higher than chance level (t(54) = 2.2; p < 0.04), whereas the accuracy for long PTs was greatly above chance level (t(54) = 5.92; p < 0.001). Note that mean accuracy did not differ from chance level when considering only the tenth-shortest PTs (t(25) = 0.5; p > 0.6). Error bars show SEMs. See also Figure S3.

In experiment 3, we simultaneously recorded eye movements and event-related potentials in 12-month-old infants (n = 55) while they engaged in a paradigm allowing for the parallel measurement of behavioral indices of confidence and neural signatures of error monitoring. This allowed us to examine (1) metacognitive abilities at earlier stages of development, during which pointing cannot be used reliably, and (2) the neural mechanisms underlying these metacognitive abilities. The first-order task consisted of looking toward a face appearing briefly on the left or right side of a screen. Faces were masked and presented for durations ranging from 50 to 300 ms (see Figure 3A), so as to induce various levels of visibility [24]. After a delay, the same stimulus consistently reappeared at the same location but now as a fully visible and rewarding face. After performing their initial choice, infants could thus decide to wait for the rewarding face at the same location (i.e., keep looking), look to the other side (i.e., change their mind), or look away (i.e., give up). This measure of post-decision persistence was used to assess infants’ confidence in their initial choice.

Infants oriented toward masked faces with above chance-level accuracy (t(54) = 5.42; p < 0.001; gaze shift direction was estimated from the offset of the face; mean response times are presented in Figure S3). There was a significant modulation of performance by stimulus duration (F(1,54) = 13.87; p < 0.001; see Figure 3B), reflecting the fact that performances were above chance level for durations ranging from 200 to 300 ms (all p values < 0.05) but not for durations from 50 to 150 ms (all p values > 0.2). Therefore, subsequent analyses collapsed the 200–300 ms durations, considered the visible condition (i.e., with above chance-level accuracy: t(54) = 6.57; p < 0.001), versus the 50–150 ms durations, considered the invisible condition (i.e., with chance-level accuracy: t < 1). On the basis of previous studies revealing that faces presented above a certain perceptual threshold trigger the same neural mechanisms as those associated with perceptual consciousness in adults, we might assume that infants were conscious of the faces only in the visible condition [24, 25]. We then inspected how PTs were affected by accuracy but found no significant main effect for this contrast. Critically, however, there was a significant interaction with stimulus visibility (F(1,54) = 4.63; p < 0.04; Figure 3C), reflecting infants’ differential persistence for correct versus incorrect trials when the masked face was visible (t(54) = 2.93; p < 0.005) but not when it was invisible (t < 1). This behavioral pattern is suggestive of confidence, and extends the results of experiment 1 to younger infants (see also Figure 3D for conditional accuracy plots of accuracy depending on PTs).

**Figure 4:**
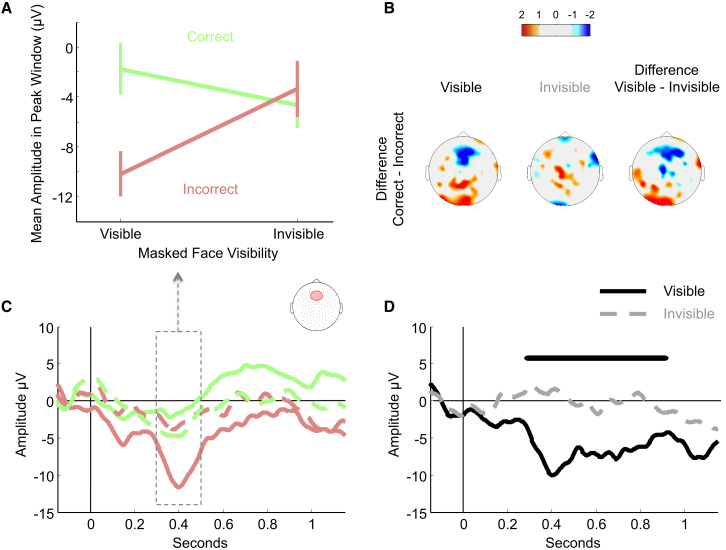
Experiment 3: Event-Related Potential Results (A) Mean amplitude in peak window depending on first-order accuracy and visibility. Error bars show SEMs. (B) Scalp topographies showing statistical significance maps (t values) of the difference between correct and incorrect trials in the peak window, computed separately for visible and invisible trials. (C) Response-locked event-related potentials (ERPs) in the fronto-central cluster depending on first-order accuracy and visibility. The dashed box and arrow show the window used to compute the mean amplitude of the peak shown in (A). (D) Difference waves for visible and invisible trials derived from response-locked ERPs by subtracting correct from incorrect trials. Bars above the time series show significant clusters with a Monte Carlo p value < 0.05. See also Figure S4.

The EEG analysis was performed with response-locked event-related potentials over fronto-central electrodes, following the standard procedure for computing an ERN [14, 15, 22]. We first collapsed all conditions to identify components of interest independent of stimulus visibility and response accuracy. Using cluster-based permutation (see the Supplemental Experimental Procedures), we identified a significant negative deflection peaking around 350 ms following the offset of the saccadic response (see Figure S4A). Crucially, the amplitude of this negativity was significantly increased for incorrect compared to correct responses in the visible condition (t(43) = 2.61; p < 0.02) but not in the invisible condition (t < 1), leading to a significant interaction (F(1,43) = 4.7; p < 0.05; see Figures 4A and 4B). Further analyses of difference waves between correct and incorrect responses confirmed this result, by revealing a significant cluster solely in the visible condition (Figures 4C and 4D). Notably, the latency of this component (around 400 ms) is much slower than what is usually observed in adults (around 100 ms). Yet, it is consistent with Berger and colleagues’ study, in which an ERN peaking around 400 ms was measured when infants observed a numerical violation in their external environment [23]. This delay of processing probably reflects the weak myelination of cortico-cortical connections achieved at the end of the first year of life [26, 27], especially in frontal areas where the ERN originates [20, 28]. Importantly, an ERN was elicited after incorrect decisions made on supraliminal stimuli but not on faces presented below the threshold of visibility. This observation mirrors a functional property of error detection in adults, for which an ERN similarly occurs for errors made on consciously reportable events but not on subliminal stimuli [14].

Thus, core neural mechanisms of error monitoring in adults are already functional at 12 months of age. Notably, when adults consciously detect their errors, the ERN is followed by a positive, slightly more posterior component (i.e., the error positivity, or Pe) [1, 15, 29]. Here we did not observe this component. This might suggest that more explicit components of error detection follow a slower developmental course. However, it is equally probable that our design was not suitable to detect this component. In particular, because infants often made a second saccade after making their first response, a Pe might have been elicited but would remain undetectable because of movement artifacts. Further studies should be especially designed to examine the presence of this component in infants. Another interesting question is whether the neural mechanisms reflected in the ERN are also involved in the computation of confidence. In adults, although the ERN amplitude varies with confidence ratings [22], the exact nature of this relationship remains unclear (e.g., see [30]). Because our persistence measure was based on looking behavior, here we were not able to elucidate the relationships between the ERN and the behavioral measure of confidence (see Figures S4B and S4C), and further studies are required to investigate this issue.

## Discussion

Our results reveal that, from early stages of development, human infants can estimate decision confidence (experiments 1 and 3), monitor their own errors (experiment 3), and use these metacognitive evaluations to guide subsequent behavior (experiment 2). This might seem surprising, because previous research relying on verbal reports documented a late emergence of the ability to provide accurate metacognitive judgments, around 3–4 years of age [3, 4, 5, 31, 32, 33]. Yet verbally reporting one’s own mental states involves more than pure metacognitive monitoring [34, 35]. Consistent with this idea, metacognition has been found to involve both explicit and implicit processing modes in adults [15, 34, 35, 36], and a few recent studies found that children can pass metacognitive tasks at younger ages when no verbal report is required [31, 33, 37]. In particular, we found that 20-month-old infants are able to ask for help non-verbally in order to avoid making errors [37]. Although this study revealed that toddlers prospectively estimate their own uncertainty, it remained unknown whether they can engage in post-decisional metacognitive evaluations. Here we show that infants are also able to retrospectively evaluate the accuracy of their own decisions. Another important issue is whether metacognition can be present in preverbal infants or, rather, emerges with the ability to give epistemic reports. Here, by testing 12-month-old infants and relying on totally implicit approaches, we show that metacognitive capacities are functional before infants start producing their first words.

What are the mechanisms underlying these competences? Our findings are in line with theoretical frameworks proposing that metacognitive sensitivity stems from simple evaluations of the quality of internal representations [1, 6, 7, 8]. These rudimentary neural computations could in principle already be present in the infant brain [33]. One possibility is thus that metacognitive operations are computed automatically as soon as infants start making decisions. By contrast, more explicit aspects of metacognition, allowing infants to effectively communicate their meta-representations to others, would emerge slowly across development.

## Author Contributions

L.G. and S.K. designed the experiments; L.G. performed research and analyzed data; S.K supervised research; L.G. and S.K. interpreted the results and wrote the paper.

## Supplemental Information

Document S1. Supplemental Experimental Procedures and Figures S1–S4

Document S2. Article plus Supplemental Information

## References

[1] Yeung N., Summerfield C. (2012). Metacognition in human decision-making: confidence and error monitoring. Philos. Trans. R. Soc. Lond. B Biol. Sci..

[2] Maniscalco B., Lau H. (2012). A signal detection theoretic approach for estimating metacognitive sensitivity from confidence ratings. Conscious. Cogn..

[3] Flavell J. (1979). Metacognition and cognitive monitoring: a new area of cognitive-developmental inquiry. Am. Psychol..

[4] Schneider W. (2008). The development of metacognitive knowledge in children and adolescents: major trends and implications for education. Mind Brain Educ..

[5] Sodian B., Thoermer C., Kristen S., Perst H., Beran M.J., Brandl J.L., Perner J., Proust J. (2012). Metacognition in infants and young children. Foundations of Metacognition.

[6] Pleskac T.J., Busemeyer J.R. (2010). Two-stage dynamic signal detection: a theory of choice, decision time, and confidence. Psychol. Rev..

[7] Kepecs A., Uchida N., Zariwala H.A., Mainen Z.F. (2008). Neural correlates, computation and behavioural impact of decision confidence. Nature.

[8] Kiani R., Shadlen M.N. (2009). Representation of confidence associated with a decision by neurons in the parietal cortex. Science.

[9] Hampton R.R. (2009). Multiple demonstrations of metacognition in nonhumans: converging evidence or multiple mechanisms?. Comp. Cogn. Behav. Rev..

[10] Smith J.D., Couchman J.J., Beran M.J. (2012). The highs and lows of theoretical interpretation in animal-metacognition research. Philos. Trans. R. Soc. Lond. B Biol. Sci..

[11] Lak A., Costa G.M., Romberg E., Koulakov A.A., Mainen Z.F., Kepecs A. (2014). Orbitofrontal cortex is required for optimal waiting based on decision confidence. Neuron.

[12] Gehring W.J., Goss B., Coles M.G.H., Meyer D.E., Donchin E. (1993). A neural system for error detection and compensation. Psychol. Sci..

[13] Fleming S.M., Frith C.D. (2014). The Cognitive Neuroscience of Metacognition.

[14] Charles L., Van Opstal F., Marti S., Dehaene S. (2013). Distinct brain mechanisms for conscious versus subliminal error detection. Neuroimage.

[15] Nieuwenhuis S., Ridderinkhof K.R., Blom J., Band G.P., Kok A. (2001). Error-related brain potentials are differentially related to awareness of response errors: evidence from an antisaccade task. Psychophysiology.

[16] Galvin S.J., Podd J.V., Drga V., Whitmore J. (2003). Type 2 tasks in the theory of signal detectability: discrimination between correct and incorrect decisions. Psychon. Bull. Rev..

[17] Fleming S.M., Lau H.C. (2014). How to measure metacognition. Front. Hum. Neurosci..

[18] Fleming S.M., Dolan R.J. (2012). The neural basis of metacognitive ability. Philos. Trans. R. Soc. Lond. B Biol. Sci..

[19] Hughes G., Yeung N. (2011). Dissociable correlates of response conflict and error awareness in error-related brain activity. Neuropsychologia.

[20] Dehaene S., Posner M.I., Tucker D.M. (1994). Localization of a neural system for error detection and compensation. Psychol. Sci..

[21] Falkenstein M., Hoormann J., Christ S., Hohnsbein J. (2000). ERP components on reaction errors and their functional significance: a tutorial. Biol. Psychol..

[22] Scheffers M.K., Coles M.G. (2000). Performance monitoring in a confusing world: error-related brain activity, judgments of response accuracy, and types of errors. J. Exp. Psychol. Hum. Percept. Perform..

[23] Berger A., Tzur G., Posner M.I. (2006). Infant brains detect arithmetic errors. Proc. Natl. Acad. Sci. USA.

[24] Gelskov S.V., Kouider S. (2010). Psychophysical thresholds of face visibility during infancy. Cognition.

[25] Kouider S., Stahlhut C., Gelskov S.V., Barbosa L.S., Dutat M., de Gardelle V., Christophe A., Dehaene S., Dehaene-Lambertz G. (2013). A neural marker of perceptual consciousness in infants. Science.

[26] Yakovlev P.I., Lecours A.R., Minkowski A. (1967). The myelogenetic cycles of regional maturation of the brain. Regional Development of the Brain in Early Life.

[27] Pujol J., Soriano-Mas C., Ortiz H., Sebastián-Gallés N., Losilla J.M., Deus J. (2006). Myelination of language-related areas in the developing brain. Neurology.

[28] Debener S., Ullsperger M., Siegel M., Fiehler K., von Cramon D.Y., Engel A.K. (2005). Trial-by-trial coupling of concurrent electroencephalogram and functional magnetic resonance imaging identifies the dynamics of performance monitoring. J. Neurosci..

[29] O’Connell R.G., Dockree P.M., Bellgrove M.A., Kelly S.P., Hester R., Garavan H., Robertson I.H., Foxe J.J. (2007). The role of cingulate cortex in the detection of errors with and without awareness: a high-density electrical mapping study. Eur. J. Neurosci..

[30] Boldt A., Yeung N. (2015). Shared neural markers of decision confidence and error detection. J. Neurosci..

[31] Balcomb F.K., Gerken L. (2008). Three-year-old children can access their own memory to guide responses on a visual matching task. Dev. Sci..

[32] Ghetti S., Hembacher E., Coughlin C.A. (2013). Feeling uncertain and acting on it during the preschool years: a metacognitive approach. Child Dev. Perspect..

[33] Vo V.A., Li R., Kornell N., Pouget A., Cantlon J.F. (2014). Young children bet on their numerical skills: metacognition in the numerical domain. Psychol. Sci..

[34] Shea N., Boldt A., Bang D., Yeung N., Heyes C., Frith C.D. (2014). Supra-personal cognitive control and metacognition. Trends Cogn. Sci..

[35] Proust J., Beran M.J., Brandl J.L., Perner J., Proust J. (2012). Metacognition and mindreading: one or two functions?. Foundations of Metacognition.

[36] Logan G.D., Crump M.J.C. (2010). Cognitive illusions of authorship reveal hierarchical error detection in skilled typists. Science.

[37] Goupil L., Romand-Monnier M., Kouider S. (2016). Infants ask for help when they know they don’t know. Proc. Natl. Acad. Sci. USA.

