# Supplementary material for: Behavioral and Neural Indices of Metacognitive Sensitivity in Preverbal Infants
Source: Curr Biol. 2016 Nov 21;26(22):3038–45. doi: 10.1016/j.cub.2016.09.004 (PMC5130696; doi:10.1016/j.cub.2016.09.004)
Supplement: Document S2. Article plus Supplemental Information [file mmc2.pdf]

Report

Current Biology

Behavioral and Neural Indices of Metacognitive Sensitivity in Preverbal Infants

Highlights

- Preverbal infants internally monitor the accuracy of their own decisions
- Infants' post-decision persistence reflects decision confidence
- Infants can rely on decision confidence to regulate subsequent behavior
- After errors, a typical EEG marker of performance monitoring is elicited

Authors

Louise Goupil, Sid Kouider

Correspondence

lougoupil@gmail.com (L.G.),
sid.kouider@ens.fr (S.K.)

In Brief

Goupil and Kouider studied implicit behaviors and neural indices in infants and show that they have metacognition. Infants showed more post-decision persistence for correct compared to incorrect choices, evidencing an evaluation of decision confidence. After a mistake, infants elicited the same neuronal signature of error monitoring as in adults.

Goupil & Kouider, 2016, Current Biology *26*, 3038–3045
November 21, 2016 © 2016 The Authors. Published by Elsevier Ltd.
http://dx.doi.org/10.1016/j.cub.2016.09.004

CellPress

Current Biology
Report

Behavioral and Neural Indices of Metacognitive Sensitivity in Preverbal Infants

Louise Goupil[1,2,4,*] and Sid Kouider[1,3,*]
[1]Brain and Consciousness Group, CNRS, École Normale Supérieure, PSL Research University, 75005 Paris, France
[2]Ecole Doctorale Cerveau Cognition Comportement, Université Pierre et Marie Curie, 75005 Paris, France
[3]Science Division, Department of Psychology, New York University Abu Dhabi, Saadiyat Island, PO Box 129188, Abu Dhabi, UAE
[4]Lead Contact
*Correspondence: lougoupil@gmail.com (L.G.), sid.kouider@ens.fr (S.K.)
http://dx.doi.org/10.1016/j.cub.2016.09.004

SUMMARY

Humans adapt their behavior not only by observing the consequences of their actions but also by internally monitoring their performance. This capacity, termed metacognitive sensitivity [1, 2], has traditionally been denied to young children because they have poor capacities in verbally reporting their own mental states [3–5]. Yet, these observations might reflect children's limited capacities for explicit self-reports, rather than limitations in metacognition per se. Indeed, metacognitive sensitivity has been shown to reflect simple computational mechanisms [1, 6–8], and can be found in various non-verbal species [7–10]. Thus, it might be that this faculty is present early in development, although it would be discernible through implicit behaviors and neural indices rather than explicit self-reports. Here, by relying on such non-verbal indices, we show that 12- and 18-month-old infants internally monitor the accuracy of their own decisions. At the behavioral level, infants showed increased persistence in their initial choice after making a correct as compared to an incorrect response, evidencing an appropriate evaluation of decision confidence. Moreover, infants were able to use decision confidence adaptively to either confirm their initial choice or change their mind. At the neural level, we found that a well-established electrophysiological signature of error monitoring in adults, the error-related negativity, is similarly elicited when infants make an incorrect choice. Hence, although explicit forms of metacognition mature later during childhood, infants already estimate decision confidence, monitor their errors, and use these metacognitive evaluations to regulate subsequent behavior.

RESULTS

Here we tested whether two core metacognitive processes, decision confidence and error monitoring, are already present in infancy. We developed a non-verbal procedure in which 18- and 12-month-old infants made a binary decision that allowed measuring performance at the cognitive (i.e., first-order) level. Post-decision measurements then allowed assessing infants' metacognitive (i.e., second-order) abilities. For confidence (experiments 1–3), we relied on a behavioral measure reflecting infants' willingness to search for a reward depending on the accuracy of their decision. Studies in rats suggest that similar measures of post-decision persistence reflect a metacognitive computation of confidence [7, 11]. More precisely, rats' willingness to wait for a reward can be accounted for by a simple model in which confidence is estimated by comparing the accumulated evidence against the decision bound [7]. Importantly, this process involves the orbito-frontal cortex, a region distinct from areas involved in first-order decisions [12] but analogous to areas involved in metacognition in humans [13]. For error detection (experiment 3), we relied on a neural marker well documented in adults. Specifically, by probing for the presence of the error-related negativity (ERN), an electroencephalography (EEG) component observed whenever subjects make an error [1, 14, 15], we tested whether the mechanisms responsible for error monitoring in adults are already functional in 12-month-old infants.

18-Month-Old Infants Monitor Decision Confidence

In experiment 1, 18-month-old infants (n = 29) saw an object being hidden in one of two opaque boxes and, after a delay, were asked to point to indicate where the object was concealed (see Figure 1A and the Supplemental Experimental Procedures). First-order performance on this task was assessed along a parametric variation of difficulty (i.e., memorizing the location of the toy for a brief or longer delay). Immediately following this choice, infants were provided with the selected box. The amount of time they were willing to search within this box before giving up was used as a measure of post-decision persistence. Importantly, persistence times (PTs) were measured in the absence of any external feedback on performance, allowing us to use this measure as a proxy for confidence [7].

Infants pointed toward the correct box with above chance-level accuracy ($t(28) = 4.9$; $p < 0.001$) but experienced more difficulty as memorization delay increased: accuracy was significantly above chance level for the shortest delay of 3 s ($t(25) = 2.81$; $p < 0.01$) but not for any of the longer delays (all p values > 0.06; Figure S1). We then examined whether infants' persistence in their initial choice correlated with accuracy. Consistent with our hypothesis, we observed that infants searched longer in the box following correct as compared to incorrect decisions ($t(28) = 2.1$; $p < 0.05$). To ensure that our

 © 2016 The Authors. Published by Elsevier Ltd.
This is an open access article under the CC BY license (http://creativecommons.org/licenses/by/4.0/).

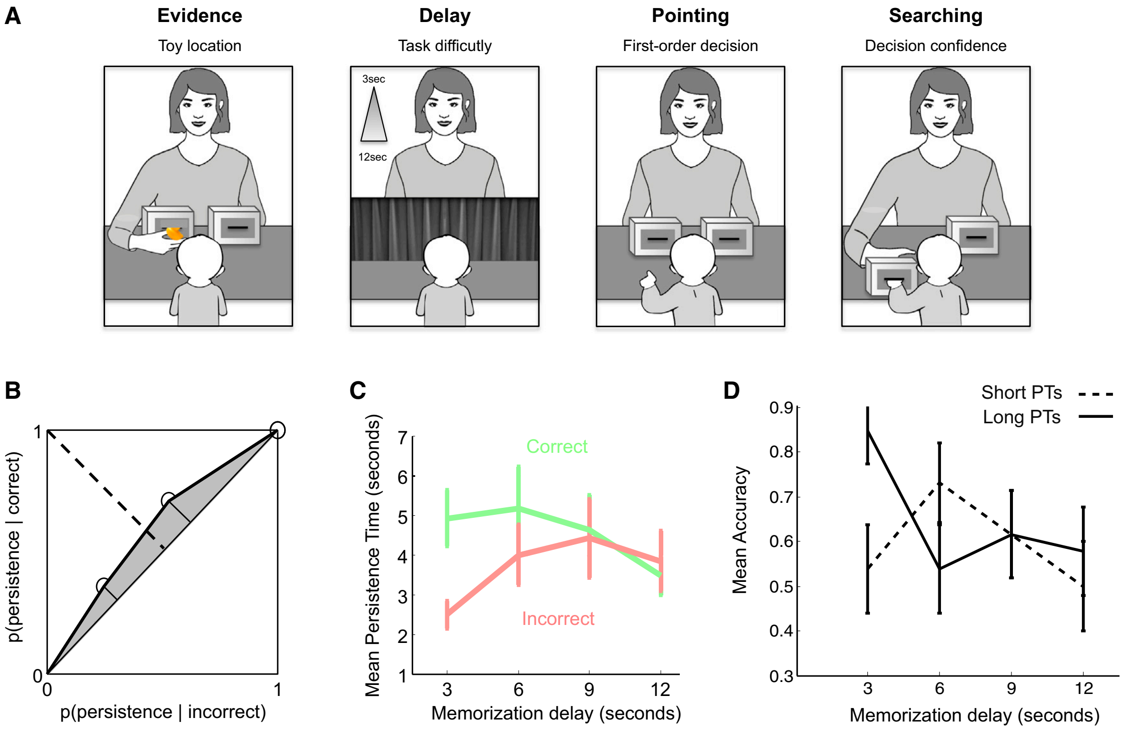

Figure 1. Experiment 1

(A) Experimental design. During the familiarization phase (four trials), the infant saw the experimenter hide a toy in one of two opaque boxes. The experimenter then asked her to point to indicate where she remembered the toy to be. As soon as the infant produced a pointing response, the selected box was pushed forward to allow retrieving the toy. The test phase (eight trials) was similar except for two aspects. First, a variable memorization delay (3, 6, 9, or 12 s) was introduced, during which a curtain was closed to occlude the boxes from the infant's view. Second, unbeknownst to the child, the toys were now out of reach during the searching period (i.e., hidden within an unreachable pocket inside the box), so as to measure infants' persistence. As soon as infants gave up searching, the experimenter recovered the toy from the box and showed it to the infant before either (1) replacing it in the box and letting the infant recover it after a correct response, or (2) starting a new trial when no response or an incorrect response was given by the infant.

(B) Mean type 2 ROC curve. Individual type 2 ROC curves were constructed by plotting the probability of searching for a certain amount of time for correct trials against the probability of searching for an equivalent amount of time for incorrect trials, cumulated across persistence time (PT) terciles. The gray area between the ROC curve and the diagonal is an estimate of the extent to which infants searched longer for correct versus incorrect trials: a type 2 ROC curve departing upward from the diagonal indicates that participants were more likely to search longer for correct over incorrect trials. The dashed line represents the minor diagonal; the solid thick line corresponds to the ROC curve; and the thin solid line corresponds to the major diagonal. Area under the ROC curve is shown in gray.

(C) Relationship between PTs and accuracy depending on task difficulty. PTs were averaged separately for correct and incorrect trials for each level of difficulty.

(D) Mean accuracy as a function of task difficulty, computed separately for short (<median) and long (>median) PTs. Although PTs did not significantly predict accuracy when all conditions were collapsed ($\chi^2 = 2.54$; $p = 0.1$), there was a significant interaction between PTs and delay ($\chi^2 = 6.34$; $p < 0.02$). In the 3-s condition, accuracy was lower for short PTs as compared to long PTs ($t(25) = 2.85$; $p < 0.01$). Importantly, the accuracy for short PTs did not differ from chance level ($t(25) = 0.38$; $p > 0.7$), whereas the accuracy for long PTs was greatly above chance ($t(25) = 4.79$; $p < 0.001$). Mean accuracy did not differ from chance level even when dividing PTs in deciles and considering only the tenth-shortest PTs ($t(25) = 0.4$; $p > 0.7$).

Error bars show SEMs. See also Figure S1.

results were not contaminated by individual biases in searching more or less in the box (e.g., liberal versus conservative profiles), we performed a complementary non-parametric and bias-free analysis [16, 17]. PT terciles were computed to approximate a three-point confidence scale and construct individual type 2 receiver operating characteristic (ROC) curves [16, 17] (Figure 1B and the Supplemental Experimental Procedures). Mean area under the type 2 ROC curves was significantly greater than chance level ($t(28) = 2.09$; $p < 0.03$), suggesting that, independent of individual searching biases, infants persisted more in their choices after a correct as compared to an incorrect choice.

Although this result suggests that infants displayed metacognitive sensitivity, a potential concern might be that infants' persistence reflected the strength of the memory trace used to perform the initial choice (i.e., a first-order decision variable), rather than a metacognitive evaluation (i.e., a second-order monitoring of the decision variable). More precisely, it might be that infants simply search longer when their memory about the toy location is strong. Given that accuracy also depends on this memory trace, a spurious correlation between accuracy and persistence might also be expected in this case. Our manipulation of the memorization delay was specifically introduced to disentangle these two interpretations. Indeed, a first-order interpretation predicts a direct correlation between PTs and delay, because the memory trace allegedly fades out with time. By contrast, a metacognitive interpretation predicts that (1) persistence should depend on accuracy, as long as a reliable memory trace is available (i.e., for above chance-level performance), and

(2) that the correlation between persistence and accuracy should vanish when no reliable memory trace is available (i.e., for chance-level performance) (see [7] for a computational account). Consistent with this last interpretation, we found an interaction between delay and accuracy ($\chi^2 = 8.97$; $p < 0.03$; likelihood ratio test; mixed models were used because of missing cells; see the Supplemental Experimental Procedures) but no main effect of memorization delay ($\chi^2 = 3.16$; $p > 0.3$). This interaction was due to infants searching less following incorrect as compared to correct trials only when accuracy was above chance (i.e., for the shortest delay of 3 s; $\chi^2 = 10$; $p < 0.01$; see Figure 1C).

Another signature of confidence is that performance should fall to chance level for the lowest levels of confidence (i.e., here for the shortest PTs). Thus, we also examined accuracy as a function of PTs and memorization delay (see Figure 1D). A mixed logistic regression revealed a significant interaction ($\chi^2 = 6.34$; $p < 0.02$), reflecting the fact that PTs significantly predicted accuracy in the easy condition (i.e., at 3 s: $\chi^2 = 7.8$; $p < 0.01$), but not at any of the longer delays (all p values > 0.3). Interestingly, the intercept of the regression did not differ from chance level (estimate: -0.14 ± 0.13; $p > 0.2$). Thus, short PTs do not predict accuracy below 50% but rather correspond to chance-level performance, as would be expected for a true marker of confidence. This result also suggests that PTs reflect confidence rather than error monitoring. Indeed, for error monitoring, below chance-level accuracy would be expected for the lowest level of persistence.

Our results are thus consistent with a metacognitive interpretation according to which infants' post-decision persistence reflects an internal—second-order—evaluation of their decision (see the Supplemental Experimental Procedures for additional discussion). Another test would be to show that infants not only persist differentially in their choices depending on accuracy but also use the output of this monitoring process to adjust behavior. In other words, if we observe that infants are able to regulate subsequent choices depending on an evaluation of a first—distinct—decision, we can infer that some form of metacognitive evaluation has taken place. In experiment 2, we thus tested whether infants can rely on confidence to appropriately select a secondary action that is totally distinct from the pointing response. Whereas the first-order task was identical to experiment 1, the metacognitive task consisted of deciding whether to confirm or invalidate the initial choice (see Figure 2A). After selecting one of the two boxes by pointing, infants received, in half of the trials, a box that they could not open by themselves. In order to recover its content, infants were consequently forced to either persist in their choice by asking their caregiver to open the selected box or revise their choice by reaching toward the alternative box. We predicted that infants' choice to persist or change their mind should depend on the accuracy of their initial decision. In addition, the relationship between first-order (pointing choice accuracy) and second-order (confirming or revising their initial choice) variables should vary with the difficulty of the task (memorization delay).

18-Month-Old Infants Use Decision Confidence to Regulate Subsequent Behavior

Infants' ($n = 22$) pointing accuracy was significantly above chance level for the delay of 3 s ($t(21) = 2.9$; $p < 0.01$), but not for the delay of 12 s ($t(21) = 1.4$; $p > 0.17$) (see Figure S2). Infants always selected one of the two possible second-order actions after making a pointing response. Thus, a two-point confidence scale was obtained by dummy coding "ask for help" responses as ones (i.e., persistence) and "changes of mind" as zeros (i.e., no persistence). This persistence index was then used to construct individual type 2 ROC curves to test whether infants' choice of a second action depended on accuracy (Figure 2B). Mean area under the type 2 ROC curves was significantly greater than chance level ($t(21) = 2.26$; $p < 0.04$), showing that infants persisted more in their initial choice after correct compared to incorrect responses. Examining the impact of task difficulty on this effect revealed an interaction between delay and accuracy ($\chi^2 = 11.25$; $p < 0.001$; see Figure 2C) but no significant effect of delay ($\chi^2 = 0.48$; $p > 0.4$). This interaction reflected the fact that the impact of accuracy on the second-order measure was significant only for the shortest delay of 3 s ($\chi^2 = 13.61$; $p < 0.001$). Thus, infants' probability of asking for help versus changing their mind was determined by the accuracy of their initial choice, but only when the memorization delay allowed for above chance-level performance. These results demonstrate that infants can rely on an internal evaluation of confidence in order to regulate subsequent behavior.

Our experiments reveal behavioral indices of decision confidence in infants. Yet it remains unclear whether such metacognitive evaluations rely on the same mechanisms as those used by adults when reporting confidence. To examine further the mechanisms underlying metacognitive sensitivity in infancy, we turned to error monitoring. Indeed, the neural computations underlying this second metacognitive process are well documented in adults [1, 18]. By probing for comparable neural signatures in infants, one might thus demonstrate shared mechanisms across the two populations. A particularly interesting signature is the ERN, an event-related potential observed over fronto-central electrodes whenever subjects make an error [1, 14, 15]. Studies in human adults show that the ERN relates to post-error adjustments and error-correcting activity [12, 19] and originates in the anterior cingulate cortex [1, 20]. They also suggest that this component reflects mechanisms of performance monitoring that function by comparing the response that should have been made with the response that was actually performed [1, 14, 21, 22].

An equivalent of the ERN has been documented when 6- and 9-month-old infants perceive an unsuspected event in their environment (i.e., a conflict between expected and actual events) [23]. More precisely, this component was evoked by simple numerical violations, and thus was driven by sensory information in the external environment. An important question is whether an ERN can be generated in infants in response to their own error (i.e., following a conflict between required and actual responses) [22]. This finding would demonstrate that infants monitor their own errors in the absence of any external feedback, by relying on internal, metacognitive computations.

Behavioral Indices of Decision Confidence and Neural Signatures of Error Monitoring in 12-Month-Old Infants

In experiment 3, we simultaneously recorded eye movements and event-related potentials in 12-month-old infants ($n = 55$) while they engaged in a paradigm allowing for the parallel

CellPress

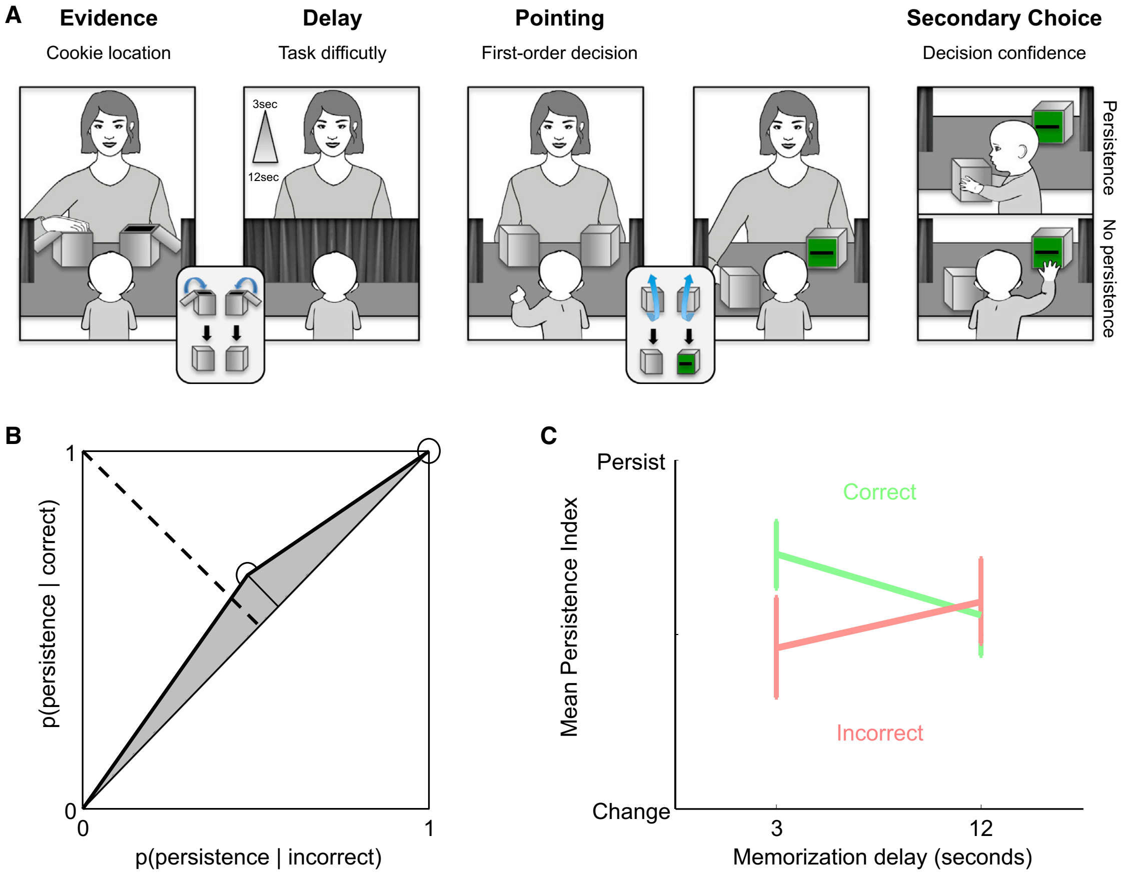

Figure 2. Experiment 2

(A) Experimental design. The two boxes contained a lid, which could only be opened by an adult. Although one of them had a slit such that infants were able to directly reach for its content (unsealed box), there was no slit in the other box, rendering its content unreachable without the help of an adult (sealed box). Importantly, both boxes looked identical from the infants' point of view, such that they could not know which box they were selecting (sealed versus unsealed box). During the familiarization phase (four trials), infants saw the experimenter hide a biscuit in one of two boxes. The experimenter then asked them to point to indicate where they remembered the biscuit to be. As soon as infants produced a pointing response, the selected box was pushed forward. During the first two trials, the biscuit was hidden in the unsealed box, so infants could directly recover it. During the last two trials, the biscuit was hidden in the sealed box, so as to teach infants to ask their caregiver to open it for them. The test phase (eight trials) was similar to the familiarization phase except for two elements. First, a variable memorization delay (3 or 12 s) was introduced. Second, the biscuits were now hidden half of the time in the unsealed box and the other half in the sealed box. Importantly, infants selected the sealed and unsealed boxes equally often ($t(21) = 0.3$; $p > 0.7$), showing that they could not discriminate the two boxes before pointing. Selection of the sealed box forced infants to either confirm their initial choice by asking their caregiver to open it or invalidate their initial choice by turning to the alternative box, whose content was directly reachable.

(B) Mean type 2 ROC curve. Individual type 2 ROC curves were constructed by plotting the probability of producing one type of secondary response for correct trials against the probability of producing the same type of secondary response for incorrect trials.

(C) Relationship between persistence and accuracy depending on task difficulty. A persistence index was obtained for each subject by coding secondary actions in a binary fashion, with zeros corresponding to changes of mind (i.e., no persistence) and ones corresponding to asking for help (i.e., persistence). Persistence indices were then averaged separately for correct and incorrect trials, per levels of difficulty, for each participant. Error bars show SEMs.

See also Figure S2.

measurement of behavioral indices of confidence and neural signatures of error monitoring. This allowed us to examine (1) metacognitive abilities at earlier stages of development, during which pointing cannot be used reliably, and (2) the neural mechanisms underlying these metacognitive abilities. The first-order task consisted of looking toward a face appearing briefly on the left or right side of a screen. Faces were masked and presented for durations ranging from 50 to 300 ms (see Figure 3A), so as to induce various levels of visibility [24]. After a delay, the same stimulus consistently reappeared at the same location but now as a fully visible and rewarding face. After performing their initial choice, infants could thus decide to wait for the rewarding face at the same location (i.e., keep looking), look to the other side (i.e., change their mind), or look away (i.e., give up). This measure of post-decision persistence was used to assess infants' confidence in their initial choice.

Infants oriented toward masked faces with above chance-level accuracy ($t(54) = 5.42$; $p < 0.001$; gaze shift direction was estimated from the offset of the face; mean response times are presented in Figure S3). There was a significant modulation of performance by stimulus duration ($F(1,54) = 13.87$; $p < 0.001$; see Figure 3B), reflecting the fact that performances were above

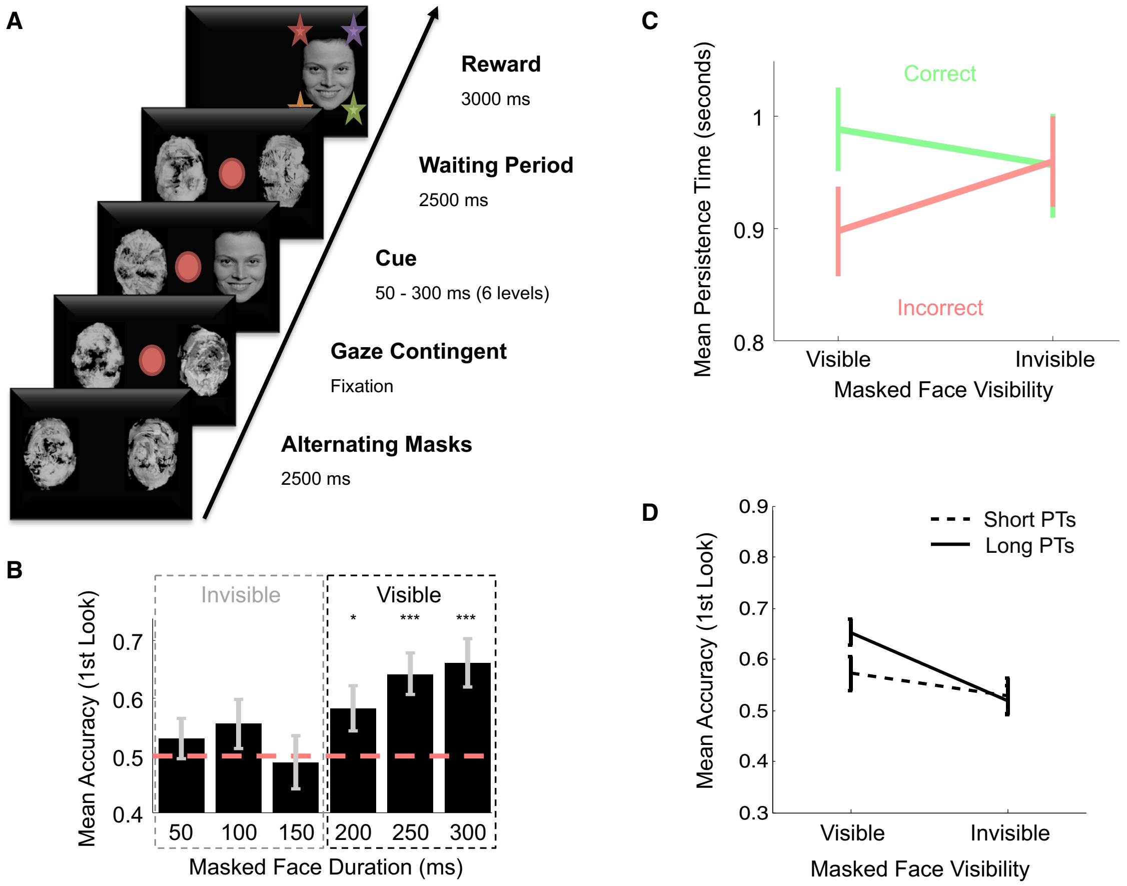

Figure 3. Experiment 3

(A) Experimental design. EEG and eye movements were recorded while infants were presented with a masked-face detection paradigm. As soon as the infant looked at the central fixation (red circle), a face was presented for variable durations (50–300 ms in steps of 50 ms) to the left or right side of the screen. A waiting period then followed, in which nothing but backward masks and the fixation remained on the screen. During this period, the first look toward one or the other side of the screen was taken as the first-order response, and the consecutive time the infant was willing to wait in the selected frame after the initial saccade was taken as post-decision persistence. Following the waiting period, the face reappeared on the same side for 3 s, now as a reward (i.e., along with music and blinking, multi-colored stars).

(B) Mean accuracy of first looks depending on the duration of the masked face. The red dashed line represents chance level. *$p < 0.05$; ***$p < 0.001$.

(C) Relationship between PTs and first-order accuracy depending on visibility. PTs were averaged separately for correct and incorrect trials for each level of visibility.

(D) Mean accuracy as a function of task difficulty, computed separately for short (<median) and long (>median) PTs. There was a marginal interaction between PTs and task difficulty ($F(1,54) = 3.7$; $p = 0.06$), reflecting the fact that PTs significantly predicted accuracy in the visible ($F(1,54) = 6$; $p < 0.02$) but not in the invisible condition ($p > 0.7$). Post hoc tests on visible trials further revealed that accuracy was marginally lower for short PTs as compared to long PTs ($t(54) = 1.97$; $p = 0.06$), and that the accuracy for short PTs remained higher than chance level ($t(54) = 2.2$; $p < 0.04$), whereas the accuracy for long PTs was greatly above chance level ($t(54) = 5.92$; $p < 0.001$). Note that mean accuracy did not differ from chance level when considering only the tenth-shortest PTs ($t(25) = 0.5$; $p > 0.6$).

Error bars show SEMs. See also Figure S3.

chance level for durations ranging from 200 to 300 ms (all p values < 0.05) but not for durations from 50 to 150 ms (all p values > 0.2). Therefore, subsequent analyses collapsed the 200–300 ms durations, considered the visible condition (i.e., with above chance-level accuracy: $t(54) = 6.57$; $p < 0.001$), versus the 50–150 ms durations, considered the invisible condition (i.e., with chance-level accuracy: $t < 1$). On the basis of previous studies revealing that faces presented above a certain perceptual threshold trigger the same neural mechanisms as those associated with perceptual consciousness in adults, we might assume that infants were conscious of the faces only in the visible condition [24, 25]. We then inspected how PTs were affected by accuracy but found no significant main effect for this contrast. Critically, however, there was a significant interaction with stimulus visibility ($F(1,54) = 4.63$; $p < 0.04$; Figure 3C), reflecting infants' differential persistence for correct versus incorrect trials when the masked face was visible ($t(54) = 2.93$; $p < 0.005$) but not when it was invisible ($t < 1$). This behavioral pattern is suggestive of confidence, and extends the results of experiment 1 to younger infants (see also Figure 3D for conditional accuracy plots of accuracy depending on PTs).

The EEG analysis was performed with response-locked event-related potentials over fronto-central electrodes, following the standard procedure for computing an ERN [14, 15, 22]. We first

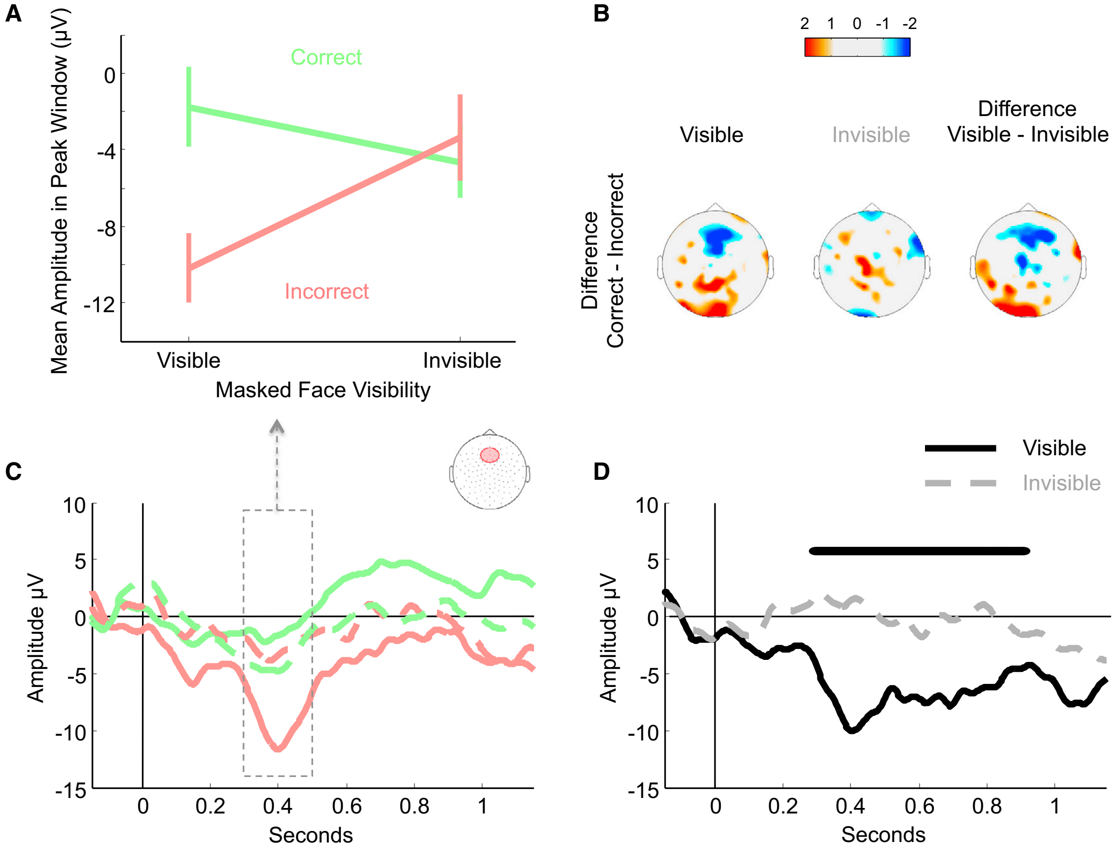

Figure 4. Experiment 3: Event-Related Potential Results

(A) Mean amplitude in peak window depending on first-order accuracy and visibility. Error bars show SEMs.

(B) Scalp topographies showing statistical significance maps (t values) of the difference between correct and incorrect trials in the peak window, computed separately for visible and invisible trials.

(C) Response-locked event-related potentials (ERPs) in the fronto-central cluster depending on first-order accuracy and visibility. The dashed box and arrow show the window used to compute the mean amplitude of the peak shown in (A).

(D) Difference waves for visible and invisible trials derived from response-locked ERPs by subtracting correct from incorrect trials. Bars above the time series show significant clusters with a Monte Carlo p value < 0.05.

See also Figure S4.

collapsed all conditions to identify components of interest independent of stimulus visibility and response accuracy. Using cluster-based permutation (see the Supplemental Experimental Procedures), we identified a significant negative deflection peaking around 350 ms following the offset of the saccadic response (see Figure S4A). Crucially, the amplitude of this negativity was significantly increased for incorrect compared to correct responses in the visible condition ($t(43) = 2.61$; $p < 0.02$) but not in the invisible condition ($t < 1$), leading to a significant interaction ($F(1,43) = 4.7$; $p < 0.05$; see Figures 4A and 4B). Further analyses of difference waves between correct and incorrect responses confirmed this result, by revealing a significant cluster solely in the visible condition (Figures 4C and 4D). Notably, the latency of this component (around 400 ms) is much slower than what is usually observed in adults (around 100 ms). Yet, it is consistent with Berger and colleagues' study, in which an ERN peaking around 400 ms was measured when infants observed a numerical violation in their external environment [23]. This delay of processing probably reflects the weak myelination of cortico-cortical connections achieved at the end of the first year of life [26, 27], especially in frontal areas where the ERN originates [20, 28]. Importantly, an ERN was elicited after incorrect decisions made on supraliminal stimuli but not on faces presented below the threshold of visibility. This observation mirrors a functional property of error detection in adults, for which an ERN similarly occurs for errors made on consciously reportable events but not on subliminal stimuli [14].

Thus, core neural mechanisms of error monitoring in adults are already functional at 12 months of age. Notably, when adults consciously detect their errors, the ERN is followed by a positive, slightly more posterior component (i.e., the error positivity, or Pe) [1, 15, 29]. Here we did not observe this component. This might suggest that more explicit components of error detection follow a slower developmental course. However, it is equally probable that our design was not suitable to detect this component. In particular, because infants often made a second saccade after making their first response, a Pe might have been elicited but would remain undetectable because of movement artifacts. Further studies should be especially designed to examine the presence of this component in infants. Another interesting

question is whether the neural mechanisms reflected in the ERN are also involved in the computation of confidence. In adults, although the ERN amplitude varies with confidence ratings [22], the exact nature of this relationship remains unclear (e.g., see [30]). Because our persistence measure was based on looking behavior, here we were not able to elucidate the relationships between the ERN and the behavioral measure of confidence (see Figures S4B and S4C), and further studies are required to investigate this issue.

DISCUSSION

Our results reveal that, from early stages of development, human infants can estimate decision confidence (experiments 1 and 3), monitor their own errors (experiment 3), and use these metacognitive evaluations to guide subsequent behavior (experiment 2). This might seem surprising, because previous research relying on verbal reports documented a late emergence of the ability to provide accurate metacognitive judgments, around 3–4 years of age [3–5, 31–33]. Yet verbally reporting one's own mental states involves more than pure metacognitive monitoring [34, 35]. Consistent with this idea, metacognition has been found to involve both explicit and implicit processing modes in adults [15, 34–36], and a few recent studies found that children can pass metacognitive tasks at younger ages when no verbal report is required [31–33, 37]. In particular, we found that 20-month-old infants are able to ask for help non-verbally in order to avoid making errors [37]. Although this study revealed that toddlers prospectively estimate their own uncertainty, it remained unknown whether they can engage in post-decisional metacognitive evaluations. Here we show that infants are also able to retrospectively evaluate the accuracy of their own decisions. Another important issue is whether metacognition can be present in preverbal infants or, rather, emerges with the ability to give epistemic reports. Here, by testing 12-month-old infants and relying on totally implicit approaches, we show that metacognitive capacities are functional before infants start producing their first words.

What are the mechanisms underlying these competences? Our findings are in line with theoretical frameworks proposing that metacognitive sensitivity stems from simple evaluations of the quality of internal representations [1, 6–8]. These rudimentary neural computations could in principle already be present in the infant brain [33]. One possibility is thus that metacognitive operations are computed automatically as soon as infants start making decisions. By contrast, more explicit aspects of metacognition, allowing infants to effectively communicate their meta-representations to others, would emerge slowly across development.

SUPPLEMENTAL INFORMATION

Supplemental Information includes Supplemental Experimental Procedures and four figures and can be found with this article online at http://dx.doi.org/10.1016/j.cub.2016.09.004.

AUTHOR CONTRIBUTIONS

L.G. and S.K. designed the experiments; L.G. performed research and analyzed data; S.K supervised research; L.G. and S.K. interpreted the results and wrote the paper.

ACKNOWLEDGMENTS

This research was supported by funding from the European Research Council (to S.K.). We thank J. Sackur and M. Romand-Monnier for discussions on the manuscript, T. Andrillon and L. Barbosa for discussions and support with EEG analysis, A.-C. Fievet and C. Girard for support with data collection, M. Romand-Monnier for creating the design caption, and F. Goffinet (Hôpital Cochin) for his support of our research. This study was approved by the local ethical committee.

Received: April 19, 2016
Revised: July 18, 2016
Accepted: September 6, 2016
Published: October 20, 2016

REFERENCES

1. Yeung, N., and Summerfield, C. (2012). Metacognition in human decision-making: confidence and error monitoring. Philos. Trans. R. Soc. Lond. B Biol. Sci. *367*, 1310–1321.
2. Maniscalco, B., and Lau, H. (2012). A signal detection theoretic approach for estimating metacognitive sensitivity from confidence ratings. Conscious. Cogn. *21*, 422–430.
3. Flavell, J. (1979). Metacognition and cognitive monitoring: a new area of cognitive-developmental inquiry. Am. Psychol. *34*, 906–911.
4. Schneider, W. (2008). The development of metacognitive knowledge in children and adolescents: major trends and implications for education. Mind Brain Educ. *2*, 114–121.
5. Sodian, B., Thoermer, C., Kristen, S., and Perst, H. (2012). Metacognition in infants and young children. In Foundations of Metacognition, M.J. Beran, J.L. Brandl, J. Perner, and J. Proust, eds. (Oxford University Press), pp. 119–133.
6. Pleskac, T.J., and Busemeyer, J.R. (2010). Two-stage dynamic signal detection: a theory of choice, decision time, and confidence. Psychol. Rev. *117*, 864–901.
7. Kepecs, A., Uchida, N., Zariwala, H.A., and Mainen, Z.F. (2008). Neural correlates, computation and behavioural impact of decision confidence. Nature *455*, 227–231.
8. Kiani, R., and Shadlen, M.N. (2009). Representation of confidence associated with a decision by neurons in the parietal cortex. Science *324*, 759–764.
9. Hampton, R.R. (2009). Multiple demonstrations of metacognition in nonhumans: converging evidence or multiple mechanisms? Comp. Cogn. Behav. Rev. *4*, 17–28.
10. Smith, J.D., Couchman, J.J., and Beran, M.J. (2012). The highs and lows of theoretical interpretation in animal-metacognition research. Philos. Trans. R. Soc. Lond. B Biol. Sci. *367*, 1297–1309.
11. Lak, A., Costa, G.M., Romberg, E., Koulakov, A.A., Mainen, Z.F., and Kepecs, A. (2014). Orbitofrontal cortex is required for optimal waiting based on decision confidence. Neuron *84*, 190–201.
12. Gehring, W.J., Goss, B., Coles, M.G.H., Meyer, D.E., and Donchin, E. (1993). A neural system for error detection and compensation. Psychol. Sci. *4*, 385–390.
13. Fleming, S.M., and Frith, C.D. (2014). The Cognitive Neuroscience of Metacognition (Springer).
14. Charles, L., Van Opstal, F., Marti, S., and Dehaene, S. (2013). Distinct brain mechanisms for conscious versus subliminal error detection. Neuroimage *73*, 80–94.
15. Nieuwenhuis, S., Ridderinkhof, K.R., Blom, J., Band, G.P., and Kok, A. (2001). Error-related brain potentials are differentially related to awareness of response errors: evidence from an antisaccade task. Psychophysiology *38*, 752–760.
16. Galvin, S.J., Podd, J.V., Drga, V., and Whitmore, J. (2003). Type 2 tasks in the theory of signal detectability: discrimination between correct and incorrect decisions. Psychon. Bull. Rev. *10*, 843–876.

17. Fleming, S.M., and Lau, H.C. (2014). How to measure metacognition. Front. Hum. Neurosci. *8*, 443.
18. Fleming, S.M., and Dolan, R.J. (2012). The neural basis of metacognitive ability. Philos. Trans. R. Soc. Lond. B Biol. Sci. *367*, 1338–1349.
19. Hughes, G., and Yeung, N. (2011). Dissociable correlates of response conflict and error awareness in error-related brain activity. Neuropsychologia *49*, 405–415.
20. Dehaene, S., Posner, M.I., and Tucker, D.M. (1994). Localization of a neural system for error detection and compensation. Psychol. Sci. *5*, 303–305.
21. Falkenstein, M., Hoormann, J., Christ, S., and Hohnsbein, J. (2000). ERP components on reaction errors and their functional significance: a tutorial. Biol. Psychol. *51*, 87–107.
22. Scheffers, M.K., and Coles, M.G. (2000). Performance monitoring in a confusing world: error-related brain activity, judgments of response accuracy, and types of errors. J. Exp. Psychol. Hum. Percept. Perform. *26*, 141–151.
23. Berger, A., Tzur, G., and Posner, M.I. (2006). Infant brains detect arithmetic errors. Proc. Natl. Acad. Sci. USA *103*, 12649–12653.
24. Gelskov, S.V., and Kouider, S. (2010). Psychophysical thresholds of face visibility during infancy. Cognition *114*, 285–292.
25. Kouider, S., Stahlhut, C., Gelskov, S.V., Barbosa, L.S., Dutat, M., de Gardelle, V., Christophe, A., Dehaene, S., and Dehaene-Lambertz, G. (2013). A neural marker of perceptual consciousness in infants. Science *340*, 376–380.
26. Yakovlev, P.I., and Lecours, A.R. (1967). The myelogenetic cycles of regional maturation of the brain. In Regional Development of the Brain in Early Life, A. Minkowski, ed. (Blackwell), pp. 3–70.
27. Pujol, J., Soriano-Mas, C., Ortiz, H., Sebastián-Gallés, N., Losilla, J.M., and Deus, J. (2006). Myelination of language-related areas in the developing brain. Neurology *66*, 339–343.
28. Debener, S., Ullsperger, M., Siegel, M., Fiehler, K., von Cramon, D.Y., and Engel, A.K. (2005). Trial-by-trial coupling of concurrent electroencephalogram and functional magnetic resonance imaging identifies the dynamics of performance monitoring. J. Neurosci. *25*, 11730–11737.
29. O'Connell, R.G., Dockree, P.M., Bellgrove, M.A., Kelly, S.P., Hester, R., Garavan, H., Robertson, I.H., and Foxe, J.J. (2007). The role of cingulate cortex in the detection of errors with and without awareness: a high-density electrical mapping study. Eur. J. Neurosci. *25*, 2571–2579.
30. Boldt, A., and Yeung, N. (2015). Shared neural markers of decision confidence and error detection. J. Neurosci. *35*, 3478–3484.
31. Balcomb, F.K., and Gerken, L. (2008). Three-year-old children can access their own memory to guide responses on a visual matching task. Dev. Sci. *11*, 750–760.
32. Ghetti, S., Hembacher, E., and Coughlin, C.A. (2013). Feeling uncertain and acting on it during the preschool years: a metacognitive approach. Child Dev. Perspect. *7*, 160–165.
33. Vo, V.A., Li, R., Kornell, N., Pouget, A., and Cantlon, J.F. (2014). Young children bet on their numerical skills: metacognition in the numerical domain. Psychol. Sci. *25*, 1712–1721.
34. Shea, N., Boldt, A., Bang, D., Yeung, N., Heyes, C., and Frith, C.D. (2014). Supra-personal cognitive control and metacognition. Trends Cogn. Sci. *18*, 186–193.
35. Proust, J. (2012). Metacognition and mindreading: one or two functions? In Foundations of Metacognition, M.J. Beran, J.L. Brandl, J. Perner, and J. Proust, eds. (Oxford University Press), pp. 234–251.
36. Logan, G.D., and Crump, M.J.C. (2010). Cognitive illusions of authorship reveal hierarchical error detection in skilled typists. Science *330*, 683–686.
37. Goupil, L., Romand-Monnier, M., and Kouider, S. (2016). Infants ask for help when they know they don't know. Proc. Natl. Acad. Sci. USA *113*, 3492–3496.

Current Biology, Volume *26*

Supplemental Information

Behavioral and Neural Indices of Metacognitive Sensitivity in Preverbal Infants

Louise Goupil and Sid Kouider

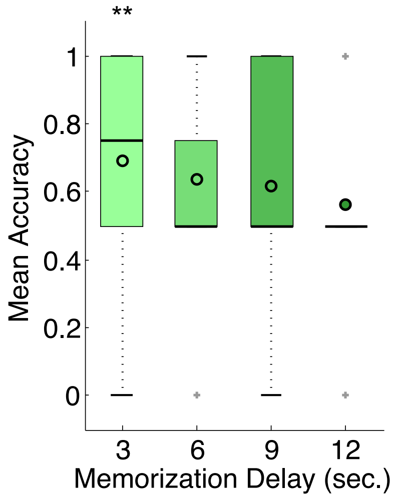

Fig. S1 (related to Fig. 1). Mean accuracy as a function of memorization delay in Experiment 1. Circles show mean accuracies; thick lines show the median; boxes delineate the 1st and 3rd quartiles; '+' signs show outlier values. The red dashed line represents the chance level. * denotes $P < 0.05$; ** denotes $P < 0.01$. The effect of task difficulty on performance does not reach significance in this experiment ($\chi^2 = 1.61$; $p = 0.2$; likelihood ratio test), probably due to a lack of power (only 2 trials per level of task difficulty). Consequently, in the subsequent experiments we increased the number of trials per infants (4 trials per levels of difficulty in experiment 2; 6 trials in experiment 3).

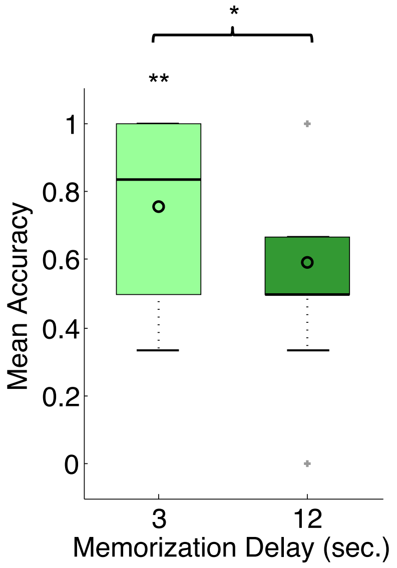

Fig. S2 (related to Fig. 2). Mean accuracy as a function of memorization delay in Experiment 2. Circles show mean accuracies; thick lines show the median; boxes delineate the 1st and 3rd quartiles; '+' signs show outlier values. The red dashed line represents the chance level. * denotes $P < 0.05$; ** denotes $P < 0.01$. There was a significant difference between the performances at 3 versus 12 seconds ($t(21) = 2.15$ - $p < 0.05$).

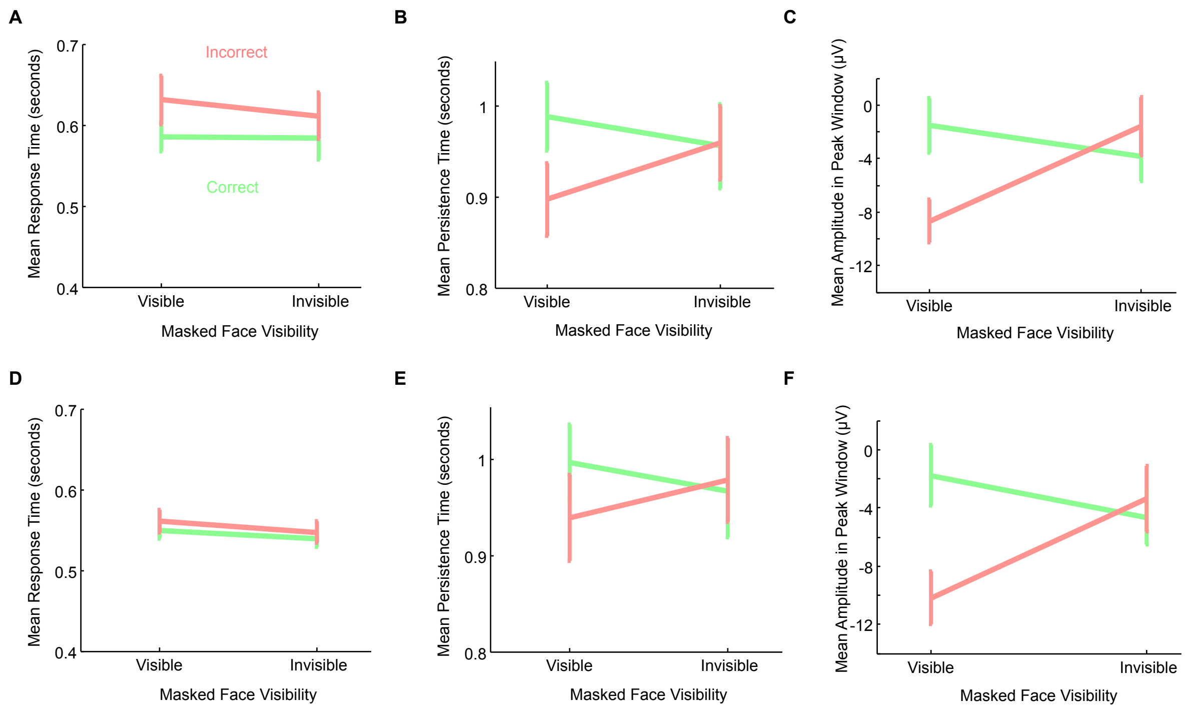

Fig. S3 (related to Fig. 3). Experiment 3. Main measures before (top row) and after (bottom row) PTs and RTs correction. (A) Response Times, (B) Persistence Times, and (C) Peak amplitude of the response-locked ERPs per conditions (accuracy and visibility). Error bars show SEMs. Although the corrections successfully resulted in cancelling any significant differences between conditions at the behavioral level (all p-values for PTs and RTs > 0.2), peak amplitude was not affected (interaction $F(1,48) = 4.2$, $p < 0.05$; difference between correct and incorrect trials for the visible condition: $t(48) = 2.48$, $p = 0.02$).

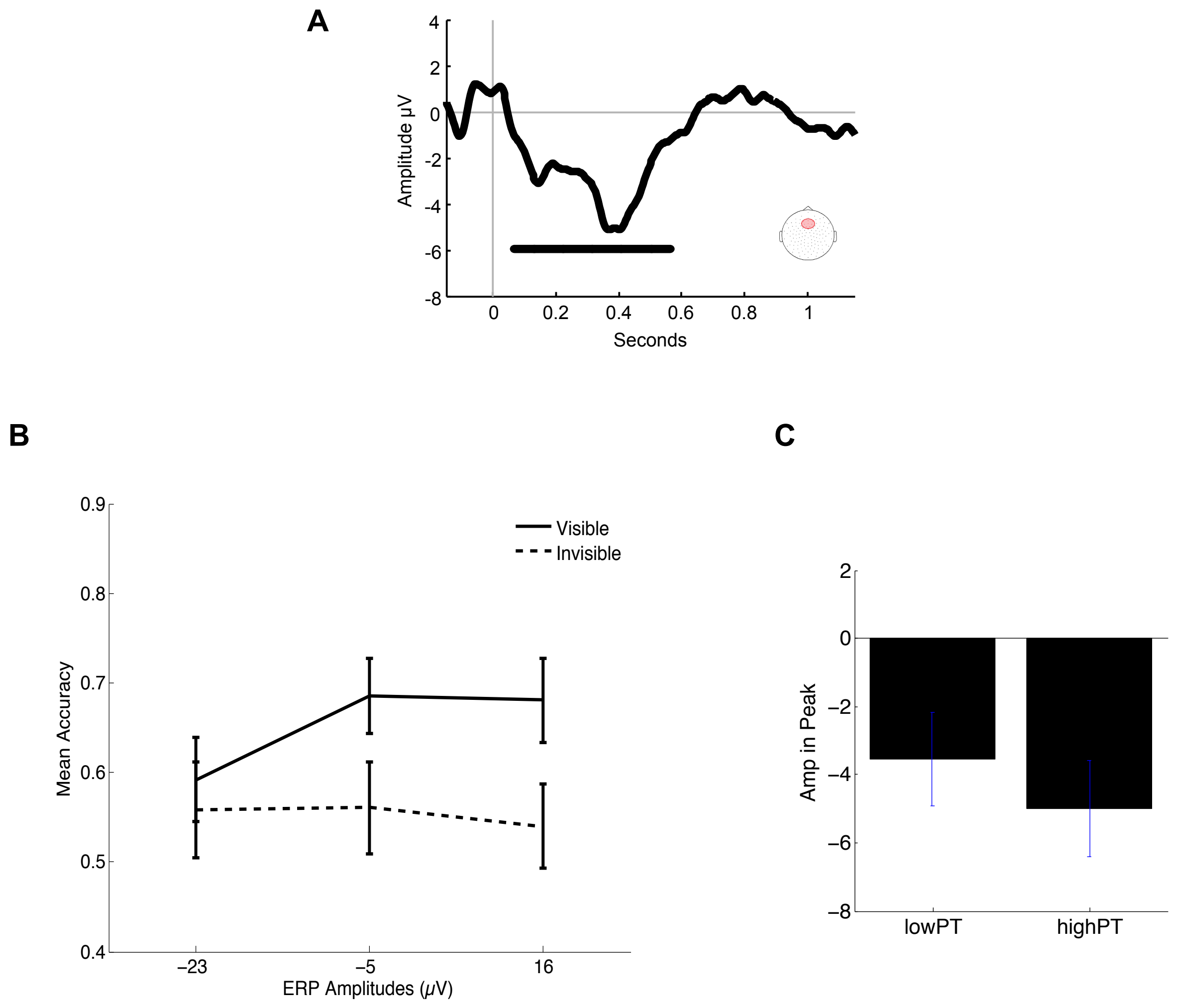

Fig. S4 (related to Fig. 4). Experiment 3. (A) Global Response-Locked ERP in the fronto-central cluster (shown in light red), obtained by collapsing all conditions. Bars above the time series show significant clusters with a Monte Carlo p value < 0.05. **(B)** Mean accuracy was computed for the 1st tercile (M = -23 μV), 2nd tercile (M = -5 μV), and 3rd tercile (M = 16 μV) of ERP amplitudes in the peak window, separately for visible (plain line) and invisible (dashed line) trials. Error bars show SEMs. A mixed logistic regression on accuracy revealed an interaction between ERP amplitude and task difficulty (χ^2 = 4.5; p = 0.04; likelihood ratio test), reflecting the fact that accuracy marginally increased with ERP amplitude in the visible (χ^2 = 2.9; p = 0.09) but not in the invisible condition (p = 0.2). In the visible condition, mean accuracy was close to chance level for the lowest bin (t(40) = 1.96 – p > 0.05) and significantly above chance at higher amplitudes (2nd tercile: t(41) = 4.4 – p < 0.001; 3rd tercile: t(41) = 3.9 – p < 0.001). In the invisible condition mean accuracy did not differ from chance level in any of the three bins (all p-values > 0.2). This analysis confirms that this ERP component reflects a mechanism of performance monitoring. Notably, these results are congruent with a study in adult participants, where strongest ERN amplitudes were observed for incorrect as well as correct responses, whenever participants thought they had made an error [S1]. **(C)** Mean ERP amplitudes were computed separately for short persistence times (low PT: below median) and long persistence times (high PT: above median). Error bars show SEMs. There was no significant difference in the amplitude of the ERP in the peak window when looking separately at high versus low persistence trials (t(43) = 0.84 - p > 0.4), even when restricting this analysis to visible trials (t(41) = 0.42 - p > 0.6). An ANOVA further confirmed that there was no significant effect of PTs on the amplitude of the ERN (F(1,43) = 0.59 – p > 0.4). This lack of relationship between PTs and ERP amplitudes is

probably due to the fact that our persistence measure relied on looking behaviour. In particular, when the infant looked for a very short time in the frame after making a saccade (i.e., for short PTs), it is likely that substantial movement artifacts were present around the timing of the ERN. It might even be that for very short PTs infants move their eyes even before an ERN has been elicited. In similar adult experiments (i.e., using the anti-saccade paradigm), participants are instructed to carry on fixating a particular location after making a response [S2]. However, this approach is not feasible in infants who cannot be instructed to remain still. Further studies relying on other persistence measures (i.e., not based of eye movements) will thus be required to investigate the relationships between decision confidence and error monitoring in infants.

Supplemental Experimental Procedures

The study was approved by the local ethical committee. All infants were healthy and born at term.

Experiment 1. Participants. Twenty-nine infants were included in the final analysis (mean age 18.16 months, age-range 17.13 – 18.90 months, 15 girls). An additional 13 infants were tested but excluded from the analysis due to fussiness before the test trials (N = 5), not performing a single pointing response in the test phase (N = 3), not performing a single pointing gesture during the experiment (N = 2), or achieving 100% first-order accuracy (N = 3).

Experimental material and procedure. Two identical boxes were constructed from white foam core (15.5 x 25.5 x 30.5 cm) and covered with black craft paper. The front of the box had a 9.5 cm by 15.5 cm opening covered by green spandex with a horizontal slit, on top of a second opaque black spandex layer with a vertical slit. Infants could thus reach into the boxes but not see inside it. Toys for the familiarization trials were two plastic toys (7 x 4 cm,) and two wooden toys (3.5 x 5 cm). Toys for the test trials were small wooden colorful shapes (3.5 x 5 cm). Toys were randomly selected on each trial. Infants sat on their caregiver's lap in front of a table (80.5 x 80.5 cm), with the experimenter sitting on the opposite side of the table. A curtain rod (80.5 x 34 cm) was installed across the table’s width, with two identical black curtains hanging on it. Toys were placed on a small trolley next to the

experimenter, and hidden from the infants view by the table. The two boxes were placed on each side of the table. Caregivers were instructed not to interact with their child and to wear opaque glasses for the entire duration of the experiment, so as not to interfere with the procedure nor the infant's behavior. A video camera placed behind the child recorded the entire session. We adapted the manual search procedure [S3] to a binary choice design with two boxes. The experiment began by introducing the boxes one by one (side counterbalanced across participants) and by saying « Look *name of the baby!* The nice box! I put my hand inside! You try! ». The experimenter then pushed the box towards the baby, encouraging her to put her hands within it. Infants then received 4 familiarization trials. On each trial, the experimenter presented one of the dedicated toys in the middle of the table before saying to the baby «Look *name of the baby!* The toy! I hide it in the box! ». In the first two trials, the box containing the toy was then pushed towards the baby such that she could recover it. In the following two trials the procedure was the same, except that after the toy had been hidden, the experimenter now said «Where is the toy? Can you show me? », while slightly pushing the two boxes forward. She then waited until the infant selected one of the two boxes by pointing or trying to reach towards one of them, before pushing the selected box forward. A verbal feedback was provided depending on the infant's response: «Yes, it is here look! » if the infant selected the correct box, or «No, it is there, look. » otherwise. In case of an incorrect choice, in this phase infants were allowed to search in the alternative box and recover the toy. In the test phase, the procedure was identical to the last two trials of the familiarization phase, except for two aspects. First, a curtain was now closed for a variable duration after the toy had been hidden in the box so as to hide the boxes from the infants' view. During this delay, the experimenter waited 3, 6, 9 or 12 seconds, while keeping her hands on the curtain rod and counting aloud to keep infant's attention on the game. Second, the toys were now surreptitiously hidden into a pocket inside the box, so that participants could never find the toys themselves. After the infant had selected one of the boxes, it was pushed forward, but subsequently recovered as soon as her hand leaved the box. The feedback was now conditional to the accuracy of the choice, with infants receiving the toy after performing a correct choice but not after giving an incorrect response, or not providing a response at all. Side (left/right) and memorization delay

(3/6/9/12 seconds) were pseudo-randomized across participants (resulting in 8 trials per participant). The experiment stopped after the infant had completed 8 trials, or went too fussy to continue.

Data processing. PTs (persistence times), RTs (response times) as well as the precise duration of the curtain's closure (memorization delay) were coded from videotapes by two independent observers (the first author and a naïve coder) blind to the experimental conditions. PTs corresponded to the period during which the first knuckles of the infants' hands were inside the box. The infant just grasping or playing with the spandex or the contours of the box was not coded as a search. RTs were defined as the time between curtains' opening and the first video frame where a response could be defined from the infants' gesture. Memorization delay was defined as the period during which the two curtains were joined, totally hiding the boxes from the infant's view. Coders agreed on 230 of the 237 responses collected (97.05%), and on 192 of the 214 PTs (89.72%, a difference was registered whenever the two codings diverged for more than 5% of the standard deviation). For this and subsequent experiments, trials with discrepancies between the two codings were recoded by a third coder blind to the conditions, and data from the naïve coder were used for all analyses – except when there was a disagreement between the two main coders, in which case data from the third coder were used. Trials with experimental errors were discarded (N = 4), as well as trials for which the recorded searching or response time exceeded 2.5 standard deviations from the mean (N = 16; computed separately for correct & incorrect trials).

Statistical analyses. In the three experiments, data were analyzed using Matlab and R (R Development Core Team, 2009) and R packages *lme4* and lmerTest [S4,S5] for mixed models analysis. Classical parametric statistics were performed (two-tailed t-tests, ANOVAs) whenever possible. However, because our contrasts involved first-order accuracy, which could not be determined a priori, we often obtained missing values within individual subjects. In those cases, mixed models were performed. We report p-values, estimated from hierarchical model comparisons using likelihood ratio tests [S6]. Persistence times (Exp. 1 & 3) were log transformed for statistical analysis, in order to

respect the assumption of normality (non-transformed data were used for display purposes). We only present models that satisfy 1) the assumption of normality (validated by visually inspecting the plots of residuals against fitted values), and 2) statistical validation (significant difference with the nested null model). To test for interactions we compared models including fixed effects versus models including fixed effects and their interaction. Similarly, to test for main effects we compared models with and without the fixed effect of interest. In experiment 1, mixed model analyses were performed because our data set contained many missing values (N subjects = 29; N conditions = 8; N observations = 178). To examine the factors impacting PTs, we performed mixed linear regressions with first-order accuracy and memorization delay as independent variables, and subject as a random factor.

Experiment 2. **Participants**. Twenty-two infants were included in the final analysis (mean age 18.10 months, age-range 17.70 – 18.50 months, 9 girls). An additional 17 infants were tested but excluded from the analysis because they did not provide pointing responses in the test phase (N = 3), managed to open the closed box themselves in the familiarization phase (N = 2), refused to eat the biscuits (N = 2), or did not provide at least 1 trial per condition (N = 10).

Experimental material and procedure. Material and apparatus were similar to experiment 1, apart from the differences listed below. Two quasi-identical boxes were constructed from cardboard (12 x 12 x 13 cm) and covered with black tape. Both boxes had a lid that could be closed by the mean of Velcro bands. Pilot testing confirmed that infants could not open the lids by themselves. The only difference between the two boxes was on the side opposite to the lid. One of the boxes was sealed: it had no opening except from the lid. The other box was unsealed: it had an additional opening on the side opposite to the lid, which was covered by green spandex with a horizontal slit so that infants could reach into the boxes while remaining unable to see inside. Biscuits were now hidden instead of toys, and infants were invited to eat them after finding them in the boxes. Infants sat on a high chair in front of a table, with the experimenter sitting on the opposite side of the table. Caregivers sat on a

chair next to their infant, and where oriented so as to face their child while looking away from the experimenter. They were thus unable to see where the biscuit was being hidden, while being able to help their child whenever the procedure required them to do so. Caregivers were instructed to open the box each time their infant handled it to them, and to stay still, neutral and quiet the rest of the time. The familiarization phase began by having the experimenter introduce the unsealed box with the lid open, allowing the infant to explore it manually, and to put her hands through the slit inside the box. The sealed box was then introduced with the lid open. After the infant explored manually the box, discovering that it had no opening at the bottom, the experimenter closed the lid and demonstrated to the caregiver how to open the lid. The lid was then closed and the box given to the parent so she could show her infant that she was competent in opening the box. Infants then received 4 familiarization trials. The first two trials were identical to the last familiarization trials of Experiment 1. Biscuits were first hidden in the unsealed box, so as to familiarize infants with the pointing procedure. In the following two trials the procedure was the same, except that now the biscuit was hidden in the sealed box, so as to familiarize infants with asking their parents to open the box for them. In the test phase, the procedure was identical to the familiarization phase, except that now the curtain was closed for a variable duration after the biscuit had been hidden in the box. Side (left/right), box containing the biscuit (unsealed/sealed) and durations (3/12 seconds) were pseudo-randomized across participants (resulting in 8 trials per participants). The experiment stopped after the infant had completed 8 trials, or went too fussy to continue.

Data processing. Pointing responses and second actions performed upon receiving the closed box were coded from videotapes by two independent observers (the first author and a naïve coder) blind to the experimental conditions. Coders agreed on 237 of the 244 pointing responses collected (97.1%), and on 118 of the 120 second actions performed by the infants (98.3%). Trials with experimental errors were discarded (N = 6).

Statistical analyses. Mixed model analyses were performed because our data set contained missing values (N subjects = 22; N conditions = 4; N observations = 83). To examine the factors impacting the secondary choice, we performed mixed logistic regressions on the persistence index (i.e. change of mind coded as 0 versus ask for help coded as 1), with first-order accuracy and memorization delay as independent variables, and subject as a random factor.

Experiment 3. **Participants.** Fifty-five infants were included in the behavioral analysis (mean age 12.14 months, age-range 11.7 – 12.7 months, 23 girls). Forty-four infants were included in the ERP analysis, as they provided at least one trial per conditions after preprocessing the EEG data. An additional 22 infants participated in the study, but were excluded from the analysis due to fussiness (n = 6), refusing to take part in the experiment (n = 3), missing values in at least one of the conditions for the behavioral analysis (n = 11), and technical issues with the setup (n = 2).

Experimental material and procedure. Images were a subset of the stimuli used in two previous studies [S7,S8]. Face stimuli consisted of 18 grayscale photos of smiling young women on a black background (15cm x 11cm). Ears and neck were removed and hairs merged into the black background. Patterns (masks) consisted of 18 grayscale images constructed by overlaying and upside-down images of a face and 3 oval objects (e.g., flowers, watches, etc.) before scrambling the layers. Both type of stimuli had the same contour size and shape, and were matched for overall luminosity and contrast. Infants sat on their caregivers' in an experimental cabin, eyes at the level of the stimuli and about 60cm from a SRS TruSurround XT screen (78 x 48 cm). Caregivers wore opaque glasses, and were asked to stay neutral during the experiment. Stimuli were presented using the Psychtoolbox for Matlab and Gaze contingency was implemented using the Eyelink toolbox for Matlab. Each trial started with the presentation of randomly chosen alternating patterns on both sides of the screen for 2500ms, with a changing rate of 500ms (see Fig. 3A). The last pair of alternating patterns (forward masks) then stayed on, as a red circle appeared at the center of the screen. If the infant looked away and seemed distracted during this phase, the experimenter pressed a button triggering a pleasant harp sound so as to attract

his attention. As soon as the infant fixated the circle for 300ms, a face appeared briefly on one side of the screen, while a pattern appeared simultaneously on the other side. This gaze contingent procedure was used to ensure that infants would always be fixating the center of the screen during the presentation of the masked face. A waiting period of 2500ms followed, during which two randomly chosen patterns (backward masks) were presented. Finally, the critical face (along with four colorful stars) reappeared as a reward/feedback on the same side, and loomed slowly for 3000ms. Side of face presentation was randomized in blocks of 6 trials. For each participant, masked face durations were randomized across 36 trials using a Latin square (6 durations, 50 to 300ms in steps of 50ms). These durations were chosen following previous studies addressing the psychophysical and neural determinants of face visibility [S7,S8]. Face stimuli were randomized across 18 trials, thus appearing twice across the experiment. To increase infants' interest, the onset of masked faces was accompanied by a bell note (e.g., "D"). The onset of reward faces was then accompanied by similar sounds creating an ensuing melody (e.g., "E" "F" "G"). Infants received 36 trials in total, unless they became too fussy to complete the entire experiment.

Eye movements recording and processing. Gaze was recorded using an Eyelink 1000 eye tracker (SR Research), and monitored online so as to automatically trigger the apparition of the critical stimulus when central fixation was detected. For the behavioral analysis, the screen was divided in three areas (left, middle and right). First-order accuracy was estimated from infants' first look towards the left or right side of the screen after seeing the masked face (i.e. during the waiting period). Persistence times were computed from the offset of the first look, as the consecutive time spent in the chosen frame (left or right). For the behavioral analysis, we excluded trials in which no response towards one or the other side of the screen was recorded, as well as trials in which infants watched the screen for less than 10% (250ms) of the waiting period [S7,S8]. We also rejected response (RTs) and persistence times (PTs) inferior to 150 ms, in order to exclude trials with anticipations, and looks crossing but not stopping in the lateral frames. For the EEG analysis, RTs and PTs were equalized across conditions, ensuring that eye movements were equally distributed across experimental

conditions (see Fig. S3). Specifically, RTs were corrected to avoid differential contamination of the response-locked activity by residual stimulus-locked activity [S2,S9]. First, RTs faster, or slower, than the 10th percentiles of the distribution were excluded. As invisible trials remained faster than visible trials after this first correction, invisible trials with RTs faster than the 15th percentile of the distribution were then excluded. A similar correction was applied to equalize PTs, in order to avoid differential contamination of the ERPs by saccadic artifacts when infants' looked away or made a saccade towards the other side of the screen. This was done by excluding the 10th fastest PTs. Although these corrections cancelled the differences between conditions at the behavioral level, the main EEG results were not affected by these manipulations (see Fig. S3). This confirms that our results were not driven by trivial differences in eye movements between experimental conditions.

EEG recording and processing. The electroencephalogram was continuously digitized at 250 Hz using a 128-electrodes HydroCel net (EGI, Eugene, OR, USA) referenced to the vertex. The signal was re-referenced to the average activity, high- and low-pass filtered (0.2Hz-20Hz), and segmented from -150ms to +1150ms around the saccadic response offset during the waiting time window. For each epoch, channels contaminated by important eye or motion artifacts (i.e. voltage exceeding +/- 400µV, or local deviations higher than 300µV over 10 samples) were rejected and their voltages automatically interpolated to the nearest electrodes. Trials with more than 35% contaminated channels were rejected. This threshold was chosen because it is widely exceeded when there is a movement, but rarely attained when the infant is quiet [S8]. We then applied a time shifted regression of EOGs on the data in order to remove ocular components from the signal, using the NoiseTools toolbox for Matlab [S10]. In addition, an artifact correction method recently developed for infants was performed [S11]. This method allows preserving epochs with aberrant local deviations, by relying on a smoothing matrix minimizing the high amplitude information (+/- 120µV) without distorting other sources contained in the signal. Finally, a baseline correction was performed from -150 to -50ms before saccade offset, and data were first averaged across response sides in order to eliminate any remaining

saccadic activity from the signal [2], before being averaged separately for each of the four conditions (correct vs. incorrect / visible vs. invisible). The mean number of artifact-free trials was 17.9 trials per infant (SEM = 1.14). On average, 2.68 trials (SEM = 0.6) were rejected per infants, and this number was similar across conditions (no effect of accuracy: $F(1,49) = 0.13$; no effect of visibility: $F(1,49) = 0.09$; no interaction: $F(1,49) = 0.59$; all p-values > 0.4). Because the ERN is well characterized in adult population[S2,S9,S12], and was previously observed in response to environmental conflict in 6- to 9-month-old infants [S13], in this study we used a region of interest approach by focusing on a cluster of fronto-central electrodes. To identify the main response-locked ERP components independently of the conditions of interest, we first collapsed all trials across conditions. In order to deal with the issue of multiple comparisons, statistical significance of the ERP across time was assessed through cluster-permutation statistics. Each cluster was constituted by the consecutive samples that passed a specified threshold of significance (p-value of 0.1; one sample ttest against zero) [S14]. For each cluster, the sum of the t-values of all the samples was then computed, and compared with the maximum cluster statistics of 1000 random permutations. Significance was assessed using a threshold monte-carlo p-value of 0.05. We identified one significant negative temporal cluster in the time window from 68ms to 560ms, peaking at 400ms (see Fig. S2). The voltage was then averaged in a temporal window of 100ms centered on the peak, and ANOVAs and post-hoc t-tests were performed to characterize the effects of first-order accuracy and visibility. Difference waves were derived for each level of visibility by subtracting correct from incorrect trials. A significant temporal cluster was found only in the visible condition, lasting from 292 to 904ms, and peaking at 404ms.

Experiment 1. Ruling out additional low-level interpretations.

Could the results be explained by a trivial fatigue effect? We verified that the observed relationship between PTs and first-order performances was not a trivial observation due to a parallel decline in PTs and performances along the experiment (i.e. fatigue effect). A linear mixed model analysis revealed

that there was indeed a main negative effect of trial rank on PTs ($\chi^2 = 3.92$, $p < 0.05$). A mixed logistic regression also revealed a similar effect on first-order accuracy ($\chi^2 = 6.37$, $p < 0.02$). This is not surprising and reflects a fatigue effect commonly found in infants' studies. Crucially however, the relationship between searching times and accuracy did not change over time ($\chi^2 = 0.75$, $p > 0.4$). This analysis indicates that the relationship between PTs and first-order accuracy found in this study cannot be reduced to a trivial effect of fatigue. On the contrary, it suggests that infants were monitoring confidence in their decisions, searching more for correct over incorrect trials from the onset of the experiment. In addition, it dismisses the possibility that the results reflect some learning of low level contingencies (e.g., infants' learning that when they search longer, or when the delay is shorter, they have a higher probability of obtaining a reward).

Could the results be explained by different levels of motivation across individual subjects? As mentioned in the main text, another concern when measuring metacognitive sensitivity is that the second-order measure can be prone to individual biases, thereby contaminating the correlation between first-order accuracy and the second-order measure. In particular, here it could have been the case that our results reflected differential levels of motivation across individuals, with highly motivated infants coincidentally showing both higher performances and longer persistence times. However, there was no correlation between individual's accuracy and overall persistence time ($r = 0.12$, $p > 0.5$), which is not consistent with this interpretation. Still, searching times were not normally distributed and varied substantially across participants (M = 4.3; SEM = 0.4 sec), reflecting individual biases to search abundantly (liberal profile) or rather scarcely (conservative profile). To ensure that our results were not contaminated by individual biases, we therefore performed a complementary non-parametric and bias-free analysis based on Receiving-Operator Characteristics (ROC) curves [S15–17]. To perform this analysis, we divided raw PTs into terciles for each participant, in order to approximate a confidence scale with 3 levels of confidence. Individual type-2 ROC curves were then constructed by plotting the cumulative probability of searching for a certain amount of time after a correct decision, against the probability of searching for the same amount of time after an incorrect

decision [S15]. Computing the area under the Type II ROC curve gives an estimation of the extent to which infants were more likely to search longer for correct versus incorrect trial: a type-2 ROC curve departing upward from the diagonal would indicate that the participant was more likely to search longer for correct over incorrect trials. Conversely, a flat ROC curve would indicate that infants searching times didn't differ between correct and incorrect trials. As reported in the main text, the area under the type-2 ROC curve was significantly different from zero, indicating that infants' metacognitive sensitivity was above chance level in this task even when taking the metacognitive bias into account. Note that there was a marginal positive correlation between type 2 ROC area and type 1 performance in this experiment (Pearson correlation: rho = 0.32 - p = 0.08), similar to what is usually observed when constructing type 2 ROC curves from confidence ratings in human adults [S15]. The results of Experiment 2 revealed a similar correlation, although it was weak and did not reach significance (rho = 0.2 - p = 0.4).

Could the results be explained by an external monitoring of Response Times? Another issue might be that instead of reflecting an internal evaluation of performances, our results are the by-product of a low-level association between persistence times and an external variable, such as response (i.e. pointing) times during the first-order task [S18]. Indeed, response times are publicly available cues that infants can monitor in the external world and that can be used to infer decision confidence without relying on an internal, metacognitive mechanism. However, there was no significant relationship between pointing times and persistence times ($p > 0.5$), ruling out the possibility that infants were simply monitoring external manifestations of their responses to adapt persistence time.

Could the results be explained by a dual mechanism account? Finally, one might argue that persistence reflects different mechanisms for the two experimental conditions (i.e., at the earliest or longer delays). Indeed, performance simply fell to the chance level for delays above 3 seconds, revealing that the memory trace was not simply weaker at longer delays, but totally absent. As a consequence, one might argue that two different mechanisms are reflected in post-decision persistence

in these two cases (i.e., baseline persistence at longer delays, versus another mechanism at shorter delays). Note that this dual mechanisms account does not necessarily invalidate the conclusion of metacognitive sensitivity at the earliest delays. Indeed, under the dual mechanism account at least two competing interpretations coexist: 1) there is metacognitive sensitivity at the earliest delay, or 2) persistence at the earliest delay simply reflects the strength of the memory trace (i.e., a cognitive representation; e.g., infants would persist more in their search when their memory trace is strong, less when the memory trace is weak). One might argue that the second hypothesis is more parsimonious because it does not entail a metacognitive redescription: we should thus reject the first hypothesis. Yet, one might also argue that a dual mechanism interpretation is less parsimonious than the metacognitive interpretation presented in our paper, because it involves 2 different mechanisms for the longest and the shortest delay. Since both positions might be argued based on the evidence presented in the first experiment alone, we decided to perform a second experiment in which persistence could not simply rely on the strength of the memory trace. Precisely, in experiment 2, our persistence measure involved making a second action: infants had to either give the box to their caregiver, or check the alternative box. In order to be optimal (i.e., maximize reward), this second action must be based on an evaluation of the accuracy of the pointing response. And contrary to experiment 1, here persistence cannot be a direct consequence of the strength of the memory (e.g., there is no reason to think that infants would change their mind more often when their memory trace is weak, or vice versa). In this sense experiment 2 rules out the first-order interpretation of the data at the earliest delay.

Experiments 1–3: Post-hoc regressions on Persistence Measures for Correct versus Incorrect trials.

In the three experiments reported in this study we observed a significant interaction between first-order accuracy and task difficulty on the persistence measure. This interaction reflected the fact that persistence tended to decrease with task difficulty for correct trials, and increase for incorrect

trials. This pattern is consistent with the idea that persistence reflects decision confidence. By contrast, this is more difficult for a memory strength account to explain. In particular, the increase in persistence in incorrect trials does not fit with the interpretation that persistence directly reflects the decision variable: if this was the case we should expect that persistence should always decrease with task difficulty.

We present below the results of post-hoc regressions of task difficulty on persistence, computed separately for correct and incorrect responses. In experiment 1, we indeed found evidence for an increase in persistence time with delay when restricting the analysis to incorrect trials. Although the linear effect in this restricted comparison did not reach significance, probably due to the few remaining data points available per infants when looking at subsets of the data, we found a significant difference when collapsing the three longer delays leading to chance level performances (i.e., 6, 9 and 12 seconds): infants searched less for incorrect trials at 3 seconds as compared to incorrect trials at longer durations (only 13 infants provide data for this analysis, $t(12) = 3.8$ - $p < 0.003$). In correct trials on the other hand, there was a marginal decrease in persistence time with delay ($\chi^2 = 3.52$; $p = 0.06$). In experiment 2, the same pattern was observed (post-hoc regression for incorrect trials: $\chi^2 = 0.8$; $p > 0.3$; correct trials: $\chi^2 = 3.53$; $p = 0.06$). In experiment 3, there was a marginal increase in PTs with task difficulty in incorrect trials ($F(1,54) = 3.6$ – $p = 0.06$), but the decrease observed in correct trials did not reach significance ($F(1,54) = 1.74$ – $p > 0.1$). Again, although these results are not significant, they are difficult for a memory trace account to explain, since under this interpretation we should always expect lower PTs for invisible as compared to visible trials.

Supplemental References

S1. Scheffers, M.K., and Coles, M.G. (2000). Performance monitoring in a confusing world: error-related brain activity, judgments of response accuracy, and types of errors. J. Exp. Psychol. Hum. Percept. Perform. *26*, 141–151.

S2. Nieuwenhuis, S., Ridderinkhof, K.R., Blom, J., Band, G.P., and Kok, A. (2001). Error-related brain potentials are differentially related to awareness of response errors: evidence from an antisaccade task. Psychophysiology *38*, 752–60.

S3. Starkey, P. (1992). The early development of numerical reasoning. Cognition *43*, 93–126.

S4. Bates, D., Maechler, M., Bolker, B., and Walker, S. (2014). lme4: linear mixed-effects models using S4 classes. R package version 1.1-6. R.

S5. Kuznetsova, A., Brockhoff, P.B., and Christensen, H.B. (2014). lmerTest: Tests for random and fixed effects for linear mixed effect models (lmer objects of lme4 package). R.

S6. Gelman, A., and Hill, J. (2007). Data analysis using regression and multilevel/hierarchical models (Cambridge University Press).

S7. Gelskov, S. V., and Kouider, S. (2010). Psychophysical thresholds of face visibility during infancy. Cognition *114*, 285–292.

S8. Kouider, S., Stahlhut, C., Gelskov, S. V, Barbosa, L.S., Dutat, M., de Gardelle, V., Christophe, A., Dehaene, S., and Dehaene-Lambertz, G. (2013). A neural marker of perceptual consciousness in infants. Science *340*, 376–80.

S9. Gehring, W.J., Goss, B., and Coles, M.G.H. (1993). A neural system for error detection and compensation. Psychol. Sci. *4*, 385–390.

S10. De Cheveigné, A., and Parra, L.C. (2014). Joint decorrelation, a versatile tool for multichannel data analysis. Neuroimage *98*, 487–505.

S11. Mourad, N., Reilly, J.P., De Bruin, H., Hasey, G., and MacCrimmon, D. (2007). A simple and fast algorithm for automatic suppression of high-amplitude artifacts in EEG data. In ICASSP, IEEE International Conference on Acoustics, Speech and Signal Processing - Proceedings.

S12. Charles, L., Van Opstal, F., Marti, S., and Dehaene, S. (2013). Distinct brain mechanisms for conscious versus subliminal error detection. Neuroimage *73*, 80–94.

S13. Berger, A., Tzur, G., and Posner, M.I. (2006). Infant brains detect arithmetic errors. Proc. Natl. Acad. Sci. U. S. A. *103*, 12649–12653.

S14. Maris, E., and Oostenveld, R. (2007). Nonparametric statistical testing of EEG- and MEG-data. J. Neurosci. Methods *164*, 177–190.

S15. Galvin, S.J., Podd, J. V, Drga, V., and Whitmore, J. (2003). Type 2 tasks in the theory of signal detectability: discrimination between correct and incorrect decisions. Psychon. Bull. Rev. *10*, 843–876.

S16. Maniscalco, B., and Lau, H. (2012). A signal detection theoretic approach for estimating metacognitive sensitivity from confidence ratings. Conscious. Cogn. *21*, 422–30.

S17. Fleming, S.M., and Lau, H.C. (2014). How to measure metacognition. Front. Hum. Neurosci. *8*, 1–9.

S18. Hampton, R.R. (2009). Multiple demonstrations of metacognition in nonhumans: Converging evidence or multiple mechanisms? Comp. Cogn. Behav. Rev. *4*, 17–28.
